# Damaged Intestinal Epithelial Integrity Linked to Microbial Translocation in Pathogenic Simian Immunodeficiency Virus Infections

**DOI:** 10.1371/journal.ppat.1001052

**Published:** 2010-08-19

**Authors:** Jacob D. Estes, Levelle D. Harris, Nichole R. Klatt, Brian Tabb, Stefania Pittaluga, Mirko Paiardini, G. Robin Barclay, Jeremy Smedley, Rhonda Pung, Kenneth M. Oliveira, Vanessa M. Hirsch, Guido Silvestri, Daniel C. Douek, Christopher J. Miller, Ashley T. Haase, Jeffrey Lifson, Jason M. Brenchley

**Affiliations:** 1 AIDS and Cancer Virus Program, SAIC-Frederick, Inc., NCI-Frederick, Frederick, Maryland, United States of America; 2 Lab of Molecular Microbiology, NIAID, NIH, Bethesda, Maryland, United States of America; 3 Lab of Pathology, NCI, NIH, Bethesda, Maryland, United States of America; 4 Department of Pathology and Laboratory of Medicine, University of Pennsylvania, Philadelphia, Pennsylvania, United States of America; 5 Centre for Regenerative Medicine, University of Edinburgh, Edinburgh, Scotland; 6 Laboratory Animal Science Program, SAIC-Frederick, Inc., NCI-Frederick, Frederick, Maryland, United States of America; 7 AdvanDX, Inc., Woburn, Massachusetts, United States of America; 8 Human Immunology Section, Vaccine Research Center, NIAID, NIH, Bethesda, Maryland, United States of America; 9 Center for Comparative Medicine, California National Primate Research Center, University of California, Davis, California, United States of America; 10 Department of Microbiology, Medical School, University of Minnesota, Minneapolis, Minnesota, United States of America; University of Zurich, Switzerland

## Abstract

The chronic phase of HIV infection is marked by pathological activation of the immune system, the extent of which better predicts disease progression than either plasma viral load or CD4^+^ T cell count. Recently, translocation of microbial products from the gastrointestinal tract has been proposed as an underlying cause of this immune activation, based on indirect evidence including the detection of microbial products and specific immune responses in the plasma of chronically HIV-infected humans or SIV-infected Asian macaques. We analyzed tissues from SIV-infected rhesus macaques (RMs) to provide direct *in situ* evidence for translocation of microbial constituents from the lumen of the intestine into the lamina propria and to draining and peripheral lymph nodes and liver, accompanied by local immune responses in affected tissues. In chronically SIV-infected RMs this translocation is associated with breakdown of the integrity of the epithelial barrier of the gastrointestinal (GI) tract and apparent inability of lamina propria macrophages to effectively phagocytose translocated microbial constituents. By contrast, in the chronic phase of SIV infection in sooty mangabeys, we found no evidence of epithelial barrier breakdown, no increased microbial translocation and no pathological immune activation. Because immune activation is characteristic of the chronic phase of progressive HIV/SIV infections, these findings suggest that increased microbial translocation from the GI tract, in excess of capacity to clear the translocated microbial constituents, helps drive pathological immune activation. Novel therapeutic approaches to inhibit microbial translocation and/or attenuate chronic immune activation in HIV-infected individuals may complement treatments aimed at direct suppression of viral replication.

## Author Summary

Persistent activation of the immune system is a hallmark of chronic HIV/SIV infections and predicts disease progression better than either plasma viral load or CD4^+^ T cell count. While the causes of immune activation during chronic infection are likely multifactorial, recent work has shown that microbial translocation is associated with immune activation. However, direct, tissue level *in vivo* evidence of translocation and the underlying mechanisms remain unclear. Here, we sought direct *in vivo* evidence of translocation, and an understanding of the timing and the underlying mechanisms. We found that in RMs, microbial translocation begins during the late acute phase of SIV infection and increases progressively during chronic infection and is associated with structural damage of the GI tract. We further discovered that immune activation is temporally and causally related to microbial translocation and by the relative inability of intestinal macrophages to bind/phagocytose translocated microbial products. In SIV-infected sooty mangabeys, however, no evidence of epithelial barrier breakdown, nor increased microbial translocation or chronic immune activation were observed. Our results provide direct evidence for microbial translocation *in vivo*, coupled with early and progressive intestinal epithelial damage, and eventual impairment of macrophage clearance associated with dissemination of microbial products and systemic immune activation.

## Introduction

Persistently elevated immune activation characterized by polyclonal B cell activation [1], increased T-cell turnover [2], increased frequencies of T cells with an activated phenotype [3], and increased levels of pro-inflammatory molecules [4] is a hallmark of disease progression in pathogenic HIV/SIV primate lentiviral infections and is a stronger predictor of disease progression than either CD4^+^ T-cell count or plasma viral load [5]. The importance of immune activation to disease progression in HIV/SIV infections is highlighted by the low levels of immune activation measured during the chronic phase of infection in natural hosts of SIV such as African green monkeys (AGMs) and Sooty mangabeys (SMs), which do not progress to AIDS [6].

While the consequences of immune activation in HIV/SIV infection are numerous and include increased numbers of activated CD4^+^ T-cell targets for the virus, attrition of the memory CD4^+^ T-cell pool and accumulation of high frequencies of terminally differentiated and exhausted memory T and B cells, the underlying mechanisms and sources of immune activation during infection are not well understood [7], [8]. Given accumulating evidence that persistent immune activation is at the heart of disease progression, understanding the mechanisms driving immune activation in chronic HIV disease will be important for the development of new adjunctive treatment strategies targeting this process. Although many factors may contribute to immune activation during chronic HIV/SIV infection, recent evidence has indicated that translocation of microbial products from the lumen of the intestine into the periphery may contribute importantly to this process [9], [10], [11], [12], [13], [14]. These microbial products can stimulate immune cells directly via pattern recognition receptors such as toll-like receptors. Indeed, immune activation related to microbial translocation occurs in other settings and has been implicated in many other pathological conditions. For example, the preconditioning chemotherapy and radiation prior to progenitor stem cell transplantation in individuals with hematological malignancies leads to damage of the tight epithelial barrier of the gastrointestinal (GI) tract resulting in microbial translocation [15]. These translocated microbial products can then stimulate the immune system, exacerbating graft versus host disease [16], [17], [18]. Microbial translocation leading to immune activation also occurs in inflammatory bowl disease [19], after invasive surgery [20], [21], and in pancreatitis [22].

While microbial translocation has been indirectly implicated in driving immune activation in chronically HIV-infected humans and SIV-infected rhesus macaques (RMs), the mechanisms underlying this phenomenon remain unclear, with enterocyte apoptosis [23], massive loss of GI tract CD4^+^ T cells [24] and/or preferential loss of GI tract Th17 cells [25], [26] all proposed as important contributing factors. Moreover, the timing of the onset of microbial translocation relative to infection has remained obscure, and direct evidence of translocation at the tissue level has been lacking. Here, using a quantitative image analysis approach to study large segments of tissue, we provide direct immunohistochemical evidence of translocation, define the timing of microbial translocation in pathogenic SIV infection of RMs and identify loss of the integrity of the intestinal epithelial barrier as a plausible mechanistic correlate of microbial translocation. The absence of translocation or associated immune activation in chronic SIV infection of SMs, which does not result in progressive disease, underscores the critical role this process plays in the pathogenesis of primate lentiviral infections and the potential value of limiting it as an approach to adjunctive therapy.

## Results

### *In vivo* detection of microbial translocation and host responses in tissues of SIV infected RMs

**Figure 1:**
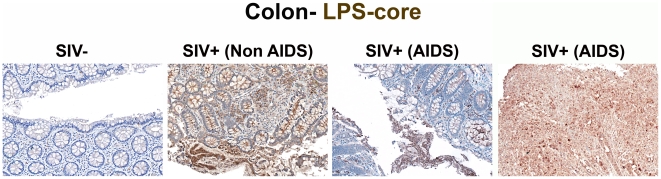
Identification of microbial translocation in large bowel of chronically SIV^+^ RMs. Representative images (200×) of unselected colon sections from SIV-uninfected and chronically SIV-infected RMs (Non-AIDS and AIDS) stained for LPS-core antigen (brown). The images show the array of microbial translocation and damage seen in our cohort that ranged from negligible (SIV- RMs) to regions of epithelial loss in our SIV^+^ Non-AIDS RMs resulting in luminal content in direct contact with the LP and increased frequency of LPS^+^ cells within the LP to more extensive epithelial damage in our SIV^+^ AIDS RMs that extended to overt ulcerations that showed significant microbial product infiltration throughout these regions; features which were absent from our SIV-uninfected RMs. Arrows point to regions of epithelial discontinuities and microbial translocation into the LP.

Initially, we sought evidence of microbial translocation by staining with a monoclonal antibody against LPS-core antigen in paraffin-embedded colon tissue sections obtained at necropsy from SIV-uninfected RMs (n = 6), RMs euthanized during early acute (n = 10) or late acute (n = 3) SIV infection, and chronically SIV-infected RMs euthanized at protocol specified endpoints (“Non-AIDS”; n = 8) or clinical endpoints (AIDS defining conditions, “AIDS”; n = 5, Table 1). Prior to and during early acute SIV infection, the LPS-core antigen-specific mAb stained only rare cells within the lamina propria (LP), but in dramatic contrast, in chronically infected animals both numerous LPS^+^ cells and abundant extracellular core LPS antigen were observed (Figures 1 and S1). Figures show a broad spectrum of microbial tanslocation and discontinuities to the structural barrier of the GI tract (discussed in detail below) that was seen in our animal cohort, from negligible (SIV-) to most severe (AIDS). Although the amount of apparently extracellular bacterial constituents within the LP varied among our chronically SIV-infected RMs; all chronically SIV-infected RMs showed readily demonstrable evidence of microbial translocation in multifocial lesions along the GI tract, findings that were absent in the SIV-uninfected animals and in early acute SIV infection (i.e. 1–10 dpi). Importantly, microbial translocation was evident in the large bowel of RMs chronically-infected with different pathogenic strains of virus (i.e. SIVmac239, SIVmac251 and SIVsmE660; Table 1), suggesting that intestinal damage leading to microbial translocation is a common feature of pathogenic SIV infections.

**Table 1:** Animals used for study.

Animal ID	Species	Virus	Days post infection	Infection Status	Major Pathologic/Clinical Findings	Opportunistic Infections	pVL^a^
595	RM	None	Uninfected	-	ND	None	NA
DBM6	RM	None	Uninfected	-	ND	None	NA
34572A	RM	None	Uninfected	-	Pneumoconiosis	None	NA
04D169	RM	None	Uninfected	-	ND	None	NA
P049	RM	None	Uninfected	-	ND	None	NA
27776^b^	RM	AT-2 SIVmac251	Uninfected	-	ND	None	NA
23053	RM	SIVmac251	1	Early Acute	Endometriosis, moderate	None	<125
30127	RM	SIVmac251	1	Early Acute	Splenomegaly, mild	None	<125
31373	RM	SIVmac251	4	Early Acute	Parathyroid Nodule	None	<125
31385	RM	SIVmac251	4	Early Acute	Gastritis	None	1.5×10^3^
30991	RM	SIVmac251	6	Early Acute	Cervical Ectopy	None	2.3×10^4^
31523	RM	SIVmac251	6	Early Acute	Generalized Lymphadenopathy, moderate	None	7.3×10^4^
26222	RM	SIVmac251	7	Early Acute	Lymphadenopathy, mild; Splenomegaly, mild; Intussusception, Ileocecal	None	2.6×10^6^
34498	RM	SIVmac251	8	Early Acute	Lymphadenopathy, generalized, marked	None	2.5×10^6^
28013	RM	SIVmac251	9	Early Acute	Lymphadenopathy, mild; Cervical ectopy	None	2.5×10^6^
24818	RM	SIVmac251	10	Early Acute	Lymphadenopathy, generalized, moderate-marked	None	9.9×10^6^
24037	RM	SIVmac239	14	Late Acute	Lymphadenopathy, generalized, marked; Splenomegaly; Hepatic Lipidosis; Endometritis, mild, Chronic	None	2.3×10^8^
27099	RM	SIVmac239	21	Late Acute	Lymphadenopathy, generalized	None	6.8×10^6^
24225	RM	SIVmac239	28	Late Acute	No significant findings	None	1.5×10^6^
551	RM	SIVmac239	168	Chronic/Non-AIDS	Lymphadenopathy, generalized, moderate; Lymphocytic portal inflammation, moderate multifocal; Pulmonary lymphoid hyperplasia, marked	None	1.0×10^6^
83I	RM	SIVmac239	168	Chronic/Non-AIDS	Renal lymphofollicular inflammation, mild focal	None	1.4×10^6^
AD09	RM	SIVmac239	161	Chronic/Non-AIDS	Lymphadenopathy, generalized, moderate; Portal hapatitis, chronic suppurative with fibrosis	None	2.7×10^6^
AY25	RM	SIVmac239	273	Chronic/Non-AIDS	Lymphadenopathy, mild; Gastritis, minimal; Colitis, mild with mucosal hyperplasia	None	3.5×10^5^ (30)^c^
AY44	RM	SIVmac239	273	Chronic/Non-AIDS	Lymphadenopathy, follicular, mild; Colitis, mild with mucosal hyperplasia; Hepatitis, mild; Nephritis, mild	None	2.010^6^ (55)^c^
R443^d^	RM	SIVmac239	391	Chronic/Non-AIDS	No apparent clinical manifestations of disease	ND	1.4×10^5^
R457^d^	RM	SIVmac239	397	Chronic/Non-AIDS	Chronic diarrhea and recurrent epistaxis	ND	8.4×10^5^
R462^d^	RM	SIVmac239	396	Chronic/Non-AIDS	Weight loss	ND	2.8×10^5^
AY99	RM	SIVmac239	213	Chronic/AIDS	Lymphadenopathy, generalized, moderate; Gastritis, mild-moderate; Colitis; mild with multifocal lymphoid follicles; Enteritis, multifocal and focally extensive with no overt OIs.	CMV meningitis, buccal Candida	1.0×10^7^
R451^e^	RM	SIVmac239	148	Chronic/AIDS	Chronic diarrhea, weight loss and cutaneous lesions; Enteritis, multifocal and focally extensive with no overt OIs.	NF	4.1×10^7f^
R475^e^	RM	SIVmac239	195	Chronic/AIDS	Chronic diarrhea, intermittent epistaxis and weight loss; Enteritis, multifocal and focally extensive with no overt OIs.	NF	7.6×10^8^
R268^e^	RM	SIVsmE660	56	Chronic/AIDS	Chronic diarrhea, Polyuria/Polydipsia and weight loss; Enteritis, multifocal and focally extensive with no overt OIs.	NF	1.4×10^9^
R437^e^	RM	SIVsmE660	184	Chronic/AIDS	Chronic diarrhea and weight loss; Enteritis, extensive with no overt OIs.	NF	7.8×10^6^
FOu	SM	None	Uninfected	-	NR	None	NA
FZr	SM	None	Uninfected	-	NR	None	NA
FAv	SM	SIVsmm	Naturally infected	Non-AIDS	NR	None	1.0×10^4g^
FBn	SM	SIVsmm	Naturally infected	Non-AIDS	NR	None	3.1×10^4g^
FBq	SM	SIVsmm	Naturally infected	Non-AIDS	NR	None	4.1×10^4g^
FFq	SM	SIVsmm	Naturally infected	Non-AIDS	NR	None	3.5×10^4g^
FKn	SM	SIVsmm	Naturally infected	Non-AIDS	NR	None	1.2×10^4g^

a Viral loads are SIV vRNA copies/mL of plasma and the detection limit of this assay was 125 copies of vRNA/ml of plasma as reported previously [53]. ^b^Animal 27776 was given i.vag. aldithriol-2 (AT-2)-inactivated SIVmac239, using the same inoculation schedule as that for the other animals inoculated i.vag. with SIVmac. This animal, had no signs of infection and was considered an uninfected control. ^c^Viral loads measured 1 week prior being placed on an ART regimen, which lasted 5 weeks. Parentheses are viral load measurements at time of euthanasia (i.e. 5 weeks post ART). ^d^Pathology reports were not available for these animals, thus only major clinical findings are reported but ^e^haematoxylin and eosin stains were examined on tissues when possible. However, we did have sufficient tissue to perform large bowel histopathological evaluation, which are also reported. ^f^Viral load measured 4 weeks before euthanasia. ^g^Viral load at time of tissue biopsy. ND, not determined. NR, not relevant, NF, none found.

**Figure 2:**
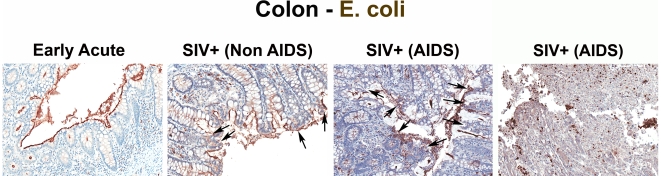
Identification of microbial translocation in large bowel of chronically SIV^+^ RMs. Representative images (200×) of colon stained with a polyclonal antibody against *E. coli* (brown) in SIV early acute infected (4 dpi) and chronically SIV-infected RMs (Non-AIDS and AIDS). Note the small regions of epithelial discontinuities in the SIV^+^ Non-AIDS RMs resulting in luminal *E. coli* ^+^ content in direct contact with the LP and increased frequency of *E. coli* ^+^ cells within the LP. Sections from two SIV^+^ AIDS RMs show the range of epithelial damage with associated microbial product infiltration, findings frequently seen in RMs with AIDS. Arrows point to regions of epithelial discontinuities and microbial translocation into the LP.

The specificity of our immunohistochemical staining directly documenting microbial translocation in gut tissue sections is supported by: (i) the remarkably low frequency of LPS^+^ cells within the LP in SIV-uninfected or RMs with early acute infection; (ii) the absence of staining with isotype-matched control antibodies in SIV-uninfected, acutely and chronically SIV-infected animals (data not shown); (iii) the lack of any evidence of non-cell associated LPS in the LP of our SIV-uninfected and early acute SIV-infected RMs, despite the abundant LPS staining in the residual luminal content of these same samples; (iv) the detection of microbial products within the LP of the colon of chronically SIV-infected RMs that were rarely seen in SIV-uninfected and early acute infected RMs, using a rabbit polyclonal antibody against *Escherichia coli* that recognizes many *E*. *coli* proteins (Figures 2 and S2), or using peptide nucleic acid fluorescent *in situ* hybridization to detect bacterial 16S RNA (data not shown).

**Figure 3:**
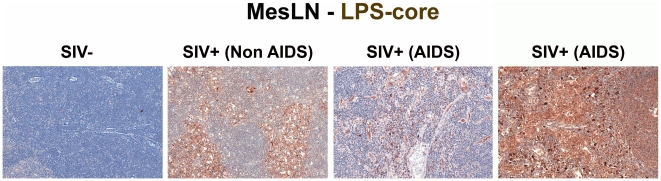
Identification of microbial translocation in gut draining MesLN of chronically SIV^+^ RMs. Representative images (200×) of the paracortical T-cell zone of MesLN stained for LPS-core antigen (brown). Note the more extensive accumulation of microbial products within the T cell zone, the T-cell inductive parenchyma of the lymphatic tissue with progressively more severe SIV infection.

**Figure 4:**
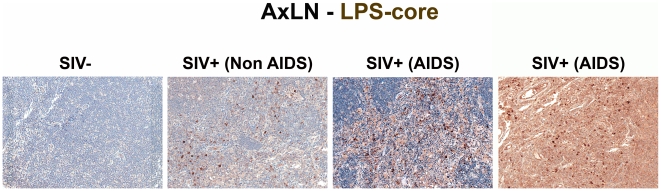
Identification of systemic microbial translocation in distant AxLN of chronically SIV^+^ RMs. Representative images (200×) of the paracortex of AxLN stained for LPS-core antigen (brown). Note the increased accumulation of microbial products within the paracortex, germinal centers, and medullary chords of the lymphatic tissue, with progressive SIV infection in chronically SIV^+^ RMs, but the paucity of LPS staining seen in SIV-uninfected RMs.

**Figure 5:**
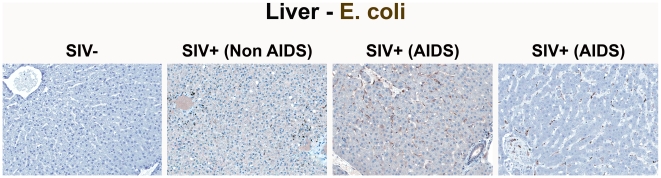
Identification of microbial translocation in the liver of chronically SIV^+^ RMs. Representative images (200×) of liver stained for*E. coli* constituents (brown). Note the increasing accumulation of microbial products near and surrounding the portal tract in chronically SIV^+^ RMs with more severe disease but lack of microbial constituent staining seen in SIV-uninfected RMs.

To relate microbial translocation in the large bowel of chronically SIV-infected RMs to systemic microbial translocation, we stained sections from lymph nodes, identifying large numbers of LPS^+^ cells within both local draining lymph nodes (mesenteric; MesLN) (Figures 3 and S3), and remote peripheral lymph nodes (axillary; AxLN) (Figures 4 and S4) of chronically SIV-infected RMs, suggesting systemic dissemination of translocated microbial constituents originating from the GI tract. Consistent with what might be expected for the anatomic site of inductive T-cell immune responses, we found immunoreactive LPS within the medullary cords and paracortex of draining MesLN as well as peripheral AxLN during the chronic stage of infection. In contrast, in SIV-uninfected or early/acute SIV-infected RMs we found only low levels of LPS^+^ cells in the gut-draining MesLN and virtually no LPS+ cells in the peripheral AxLN of (Figures 3,4 S3 and S4). The primary localization of LPS within the medullary cords and sinuses, and to a lesser extent the paracortex and germinal centers, in SIV^+^ Non-AIDS RMs is consistent with microbial products traversing from the damaged intestine via the lymphatics. The presence of LPS within the paracortex and germinal centers suggests a) antigen presenting cells which have bound microbial antigens migrate into the T cell inductive site of the LN and b) possibly microbial product-immune complex deposition on follicular dendritic cells may be occurring. However, the biological relevance of finding microbial constituents in these anatomical sites, at this point, remains unclear. Moreover, consistent with the liver's important function as a “gatekeeper” between the intestine and peripheral circulation, we also found multifocal evidence of microbial products within the liver, in regions surrounding the hepatic portal veins and tracts in chronically SIV-infected Non-AIDS RMs with more extensive staining into the lobules of the liver in chronically SIV-infected AIDS RMs, consistent with increased intestinal damage correlating with more microbial dissemination (Figure 5 and Figure S5).

Using these approaches, we identified unequivocal direct evidence of microbial constituents within the LP of the large bowel, and in the liver and lymph nodes of all chronically SIV-infected RMs studied, but not uninfected RMs. This finding was consistent across all 32 RMs studied, including SIV-uninfected RMs (n = 6); early acute SIV-infected RMs (1–10 dpi, n = 10); late acute RMs (14–28 dpi infection, n = 3); and chronically SIV-infected RMs (56–397 dpi, n = 13 (Non-AIDS and AIDS); Table 1) and indicate that microbial translocation involves infiltration of microbial products into the LP of the GI tract during the chronic phase of SIV infection of RMs.

### Quantitative image analysis of microbial translocation

**Figure 6:**
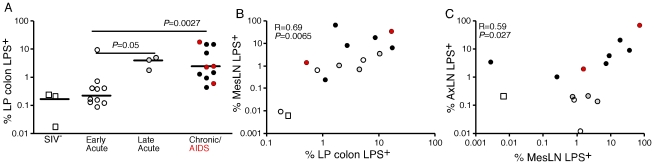
Quantitative image analysis of microbial translocation. (**A**) Levels of LPS within the LP of the colon of SIV-uninfected (white squares), early acute (white circles), late acute (gray circles), chronic Non-AIDS (black circles) and chronic AIDS (red circles) RMs. Mann-Whitney U test performed between the chronically infected (both Non-AIDS and AIDS) and early acute RMs and between late acute and early acute RMs. (**B**) Correlation between LPS levels within the LP of the colon and the mesenteric LN for animals in which we had obtained both colon and MesLN samples. Significance determined using Spearman's rank correlation coefficient and associated p-value shown. (**C**) Correlation between LPS levels within MesLN and AxLN for animals in which we had obtained both MesLN and AxLN samples. Significance determined using Spearman's rank correlation coefficient and associated P-value shown.

After demonstrating the qualitative presence of increased microbial products in the LP of large bowel, liver and lymph nodes of SIV infected RMs, we used quantitative image analysis techniques [27] to quantify the extent of microbial translocation demonstrable in high power (400×) digital scans, of tissue section whole mounts (ScanScope CS System, Aperio). Sections of colonic mucosa analyzed for each animal represented, on average, a total of 350 distinct 400× image fields per scanned tissue, providing an in-depth, systematic, assessment of segments of the GI tract that ranged from 43 to 101 linear mm of colonic epithelial lining and 16 to 39 mm^2^ of intestinal mucosal area. This comprehensive analysis approach, applied to randomly selected tissue sections, provided us with an unbiased and detailed evaluation of microbial translocation in representative sections of the GI tract that otherwise may not have been possible using conventional established tissue analysis methods. Using this approach, we found that the percent area of LP of the colonic mucosa containing LPS was significantly higher in chronically-infected RMs compared to uninfected and acutely (early) SIV-infected RMs (Figure 6A).

We used the same quantitative image analysis approach to evaluate the relationship between the microbial product burden within the LP of the colon and in draining (MesLN) and distant LN (AxLN) from the same animals, calculating the percent area of each tissue that contained LPS. We found a significant positive correlation between the amount of LPS within the LP of the colon and the amount of LPS within the corresponding draining MesLN (r = 0.69, *P* = 0.0065, Figure 6B). Moreover, we also found a significant positive correlation between LPS staining within the MesLN and within the matched AxLN (r = 0.59, *P = *0.027, Figure 6C). Taken together, these data indicate that the presence of microbial products in the peripheral lymphatic tissues is intimately linked to microbial translocation from the gut.

### Induction of immune activation: translocated microbial products co-localize with markers of immune activation

Microbial products can directly stimulate the innate immune system via interactions with Toll-like receptors (TLR) that lead to an inflammatory cascade. To evaluate the possible relationship between translocation of microbial products and inflammation we performed double-label immunohistochemical staining for microbial products and the innate proinflammatory cytokine IFNα. Reflecting the immune activation of chronic SIV infection in RMs, IFNα expression was widespread and we consistently demonstrated co-localization of IFNα and microbial products within the LP of the colon (Figure S6), and in AxLN, and MesLN (data not shown). The majority of such co-localization occurred in the absence of detectable local viral replication, as during the chronic phase of infection productively infected cells within the LP are only rarely demonstrable by *in situ* hybridization in most SIV-infected RMs that have not progressed to AIDS (data not shown). Indeed, in extensive double label studies using immunohistochemical staining for IFNα and *in situ* hybridization for SIV RNA in tissues from chronically SIV-infected Non-AIDS RMs, there was only very limited co-localization between IFNα expression and *in situ* hybridization for SIV RNA in the colon or MesLN (data not shown). The overwhelming majority of the abundant IFNα immunostaining was found relatively distant from local viral replication, but in close proximity to microbial products (data not shown). Moreover, the levels of IFNα immunostaining were significantly greater than the tissue level of SIV RNA.

To evaluate further the role of translocated microbial products in driving immune activation in chronic SIV infection, we assessed the distribution and co-localization of interleukin-18 (IL-18) and LPS in the gut-draining MesLN in SIV-uninfected and chronically SIV-infected Non-AIDS RMs (Figure S7). IL-18 is produced by activated macrophages and dendritic cells in response to microbial product stimulation [28]. We found that before SIV infection there was a basal, low-level, expression of IL-18 in all structural compartments of MesLN (i.e. B cell follicles, T-cell zone and medullary cords) and that this expression was dramatically increased in SIV infection (data not shown). Although IL-18 was up-regulated in some regions where there were no visualized microbial products, high levels of IL-18^+^ cells were always found in close proximity to LPS in MesLN (Figure S7). Consistent with a role for translocated microbial products inducing immune activation and IL-18 expression, Ahmad and colleagues recently described significantly elevated levels of IL-18 in the serum of HIV-infected/AIDS patients compared to those of HIV-seronegative healthy individuals [29]. Collectively, these data strongly suggest that microbial products, which infiltrate the LP of the GI tract, and then spread systemically, can directly stimulate the immune system and contribute to chronic immune activation.

### Timing and mechanisms of increased microbial translocation

To determine how early during infection microbial translocation occurs, we compared microbial translocation into the LP of the colon of RMs throughout the acute stage of SIV infection (1 to 28 dpi) in vaginally-challenged animals. With the exception of one animal at 8 dpi, we found only very low levels of LPS^+^ cells within the LP of RMs between 1–10 dpi, observations that were indistinguishable from SIV-uninfected animals (Figures 6 and data not shown). There was a statistically significant increase in LPS seen within the LP of animals infected for between 14 and 28 days compared to uninfected or early/acute animals and evidence of microbial translocation was detected at small foci associated with breaks in the epithelial lining (Figures 6 and data not shown). Importantly, during the acute phase of infection, areas of discontinuity in the epithelial barrier were relatively infrequent, while LPS staining in the LP appeared to increase into the late acute stage of SIV infection (14–28 dpi). Interestingly the extent of lesions and discontinuities were lower than might have been expected relative to the massive enterocyte apoptosis previously described as peaking at 14 dpi in these same animals [23]. We comment below on the possible mechanisms responsible for this dissociation.

**Figure 7:**
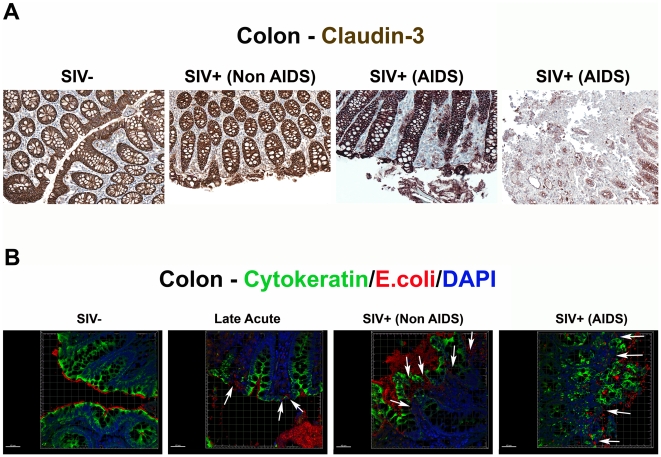
Damage to the integrity of the epithelial barrier is associated with infiltration of microbial products into the lamina propria. (**A**) Representative images (200×) of colon stained for the tight junction protein claudin-3 (brown) as a marker for intact epithelial barrier. Note the multifocal regions of the epithelial barrier that are missing in SIV^+^ RMs, allowing direct contact of luminal contents with the LP. (**B**) Confocal immunofluorescent 3-D images of colon (400×) stained for cytokeratin (epithelial cells; green), *E. coli* (red) and DAPI (nuclei; blue). Discontinuities in the epithelial barrier allowing translocation of microbial products into the LP are present in late acute SIV^+^ RMs (14 dpi) and progressively more severe in chronic Non-AIDS and chronic AIDS RMs (white arrows point to epithelial discontinuities and translocated microbial products). Scale bars  = 40 µm.

**Figure 8:**
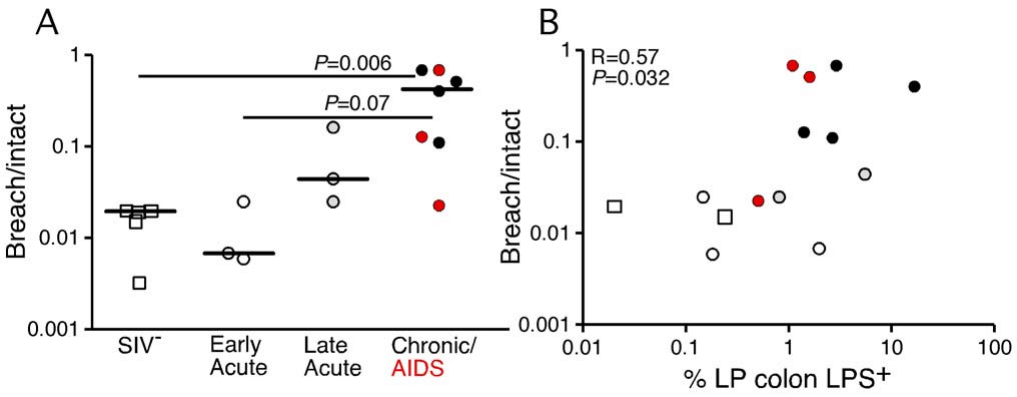
Damage to the integrity of the epithelial barrier correlates with microbial translocation. (**A**) Quantitative image analysis showing the proportion of the epithelial lining in the large bowel that is damaged (discontinuous) in SIV-uninfected (white squares), early acute (white circles), late acute (gray circles), chronic Non-AIDS (black circles) and chronic AIDS (red circles) RMs. Mann-Whitney U test performed between the chronically infected (both Non-AIDS and AIDS) and early acute RMs and between chronically infected (both Non-AIDS and AIDS) and SIV- RMs. (**B**) Statistically significant positive correlation between the proportion of epithelial damage and the level of LPS within the LP of the colon. Significance determined using Spearman's rank correlation coefficient and associated P-value shown.

Although enterocyte apoptosis subsequently decreased, even as microbial translocation increased in the late acute stage of infection, the abundant evidence of LPS^+^ within the lamina propria at 28 dpi suggested that early damage to the integrity of the epithelial lining could facilitate translocation of microbial products (data not shown). Thus, we sought to determine directly if the compromise of the integrity of the epithelial barrier is a distinguishing feature of microbial translocation during chronic SIV infection, evaluating the integrity of the epithelial barrier by staining for the tight junction protein claudin-3. A similar technique has been used in studying human samples to assess the integrity of the epithelial barrier in diseases associated with discontinuities in the GI tract [30]. Examination of the integrity of the epithelial barrier by staining for claudin-3, revealed multifocal disruptions and epithelial loss of the normally continuous, epithelial barrier in tissues from the chronic stages of infection, but not in tissues from uninfected or early acute animals (Figure 7A and Figure S8). We recognize the possible contribution that undetected opportunistic enteric pathogens may play in the continuum of epithelial damage seen in our chronically SIV-infected (AIDS) RMs, and thus show in Figures 7 and S8 two examples of end-stage RMs demonstrating this dynamic spectrum of epithelial damage from multifocal epithelial loss to severe epithelial damage and ulceration. Importantly, confocal analysis showed that disruptions in the integrity of the epithelial barrier were directly associated with translocated microbial products (Figure 7B). Furthermore, quantitative image analysis from high power entire colonic tissue section scans, confirmed that chronically-infected animals had significantly more damage to the integrity of the epithelial barrier compared to uninfected and acutely-infected RMs (Figure 8A). Moreover, the degree to which the integrity of the epithelial barrier was compromised was significantly correlated with the amount of LPS within the LP (Figure 8B; r = 0.57, P = 0.032).

**Figure 9:**
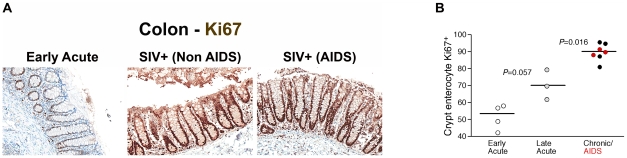
Damage to the integrity of the epithelial barrier is associated with increased enterocyte proliferation. (**A**) Representative images (200×) of colon stained for the proliferative nuclear protein Ki67 (brown). Note progressive change in frequency and location of Ki67^+^ enterocytes, from few cells at the base of crypts during early acute infection (4 dpi), with full involvement of crypts with proliferating cells in chronically infected Non-AIDS and AIDS animals. (**B**) Quantitative image analysis of Ki67 expression by colonic enterocytes in early acute (white circles), late acute (gray circles), chronic Non-AIDS (black circles) and chronic AIDS (red circles) RMs demonstrates the statistically significant increase in enterocyte proliferation in chronic infection. Mann-Whitney U test performed between the chronically infected (both Non-AIDS and AIDS) and late acute RMs and between early acute and late acute RMs.

Loss of integrity of the epithelial barrier of the GI tract would be expected to result in several host responses including polymorphonuclear neutrophil (PMN) infiltration and increased local proliferation of enterocytes within colonic crypts in an attempt to restore the integrity of the GI tract. Importantly, we observed increased levels of myeloperoxidase+ PMNs within the lamina propria of chronically SIV-infected RMs associated with damage to the epithelial barrier (Figure S9), providing strong evidence for a tissue specific response to GI epithelial damage. To assess proliferation of GI tract enterocytes we immunohistochemically stained colon tissues with a monoclonal antibody against Ki67, a cellular marker for proliferation, and performed quantitative image analysis measuring the fraction of enterocytes along the colonic crypt that were proliferating (Ki67^+^). We found increased levels of proliferating enterocytes (Ki67^+^) in chronically SIV-infected animals compared to SIV uninfected and acutely infected RMs (Figure 9A–B and Figure S10, *P* = 0.016). Moreover, there was a trend towards an increase of Ki67^+^ enterocytes in animals between 14 and 28 days post SIV infection compared to animals infected for ∼1 week (Figure 9B, *P* = 0.057). These data are consistent with early (14–28 dpi) and progressive damage to the integrity of the epithelial barrier, and indicate that one mechanism underlying microbial translocation likely involves breakdown of the structural barrier of the GI tract at a rate that exceeds enterocyte proliferation and other repair mechanisms, consistent with previous reports of abnormalities within the GI tracts of chronically HIV or SIV-infected individuals [31], [32], [33], [34], [35], [36], [37], [38]. Our findings of host responses to microbial-product infiltration into the lamina propria are consistent with previous findings of fibrosis within the lamina propria of the GI tract [39]. While our data and those of previous studies are consistent with increased discontinuity of the structural barrier of the GI tract during chronic SIV infection of RMs, we cannot exclude that the structural damage we observed by immunohistochemical analysis may be attributed to increased enterocyte turnover overall, leading to an apparent increase in structural damage to the GI tract. However, the increased levels of myeloperoxidase+ PMNs within the lamina propria of chronically SIV-infected RMs associated with observed damage to the epithelial barrier (Figure S9), strongly support our conclusion of GI epithelial damage. Regardless, these data suggest that the integrity of the structural barrier of the GI tract is significantly weakened in SIV-infected individuals leading to microbial translocation.

### Microbial translocation and phagocytosis by intestinal macrophages

**Figure 10:**
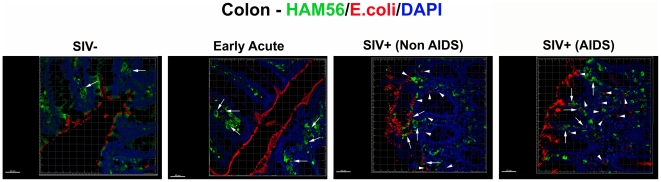
Microbial products are differentially phagocytosed in acutely and chronically SIV-infected RMs. Confocal immunofluorescent 3-D images of colon (400×) stained for HAM56 (macrophages; green), *E. coli* (red) and DAPI (nuclei; blue) showing the progressive decrease in the fraction of microbial products (*E. coli*, red) internalized by macrophages (green, highlighted by white arrows), and an increase in the proportion that were not cell associated with progressively more severe SIV infection. For specimens obtained from animals with later stages of infection, the presence of abundant HAM56+ macrophages (green) without internalized *E. coli* constituents (red) (highlighted by white arrow heads), despite extensive *E. coli* constituents present in the LP near macrophages, suggests that macrophages in chronic SIV^+^ RMs become less able to efficiently clear microbial products translocated into the LP. Rare microbial constituents are present in SIV-uninfected RMs but are virtually always found within macrophages. Scale bars  = 40 µm.

When microbial products cross the epithelial barrier under normal, physiological conditions, they are generally phagocytosed by specialized intestinal macrophages [40]. The relatively abundant and apparently non-cell associated microbial constituents we saw in the LP of our chronically SIV-infected RMs, particularly in animals with AIDS, suggested that this might result from microbial translocation in excess of the phagocytic capacity of these macrophages. This could reflect a saturation of the maximum capacity of these macrophages to phagocytose translocated microbial constituents or alternatively, might reflect a compromise of macrophage phagocytic function resulting in a net relative defect in microbial clearance and the extracellular accumulation of microbial products. To assess this, we performed confocal microscopy of GI tract tissue from SIV-uninfected and acute and chronically SIV-infected RMs to assess the localization of microbial constituents relative to GI tract macrophages (intracellular vs. extracellular). In the rare instances where microbial products were detected in the LP of SIV-uninfected RMs, they were virtually always within HAM56^+^ macrophages (Figure 10 and Figure S11). In addition, during the acute phase of infection (until 28 dpi), most microbial products were mostly found within HAM56^+^ macrophages (Figure S11), perhaps helping to explain why LPS levels in plasma are not elevated during acute infection while sCD14 levels were moderately raised [9]. Moreover, microbial products crossing the epithelial barrier at 14 dpi were virtually always found within macrophages, whereas abundant macrophages were juxtaposed to damaged regions of the colon in late acute (28 dpi) (Figure S11). Furthermore, as infection progressed into the chronic stage of disease, the frequency of macrophages negative for microbial products increased, even though abundant numbers of macrophages were present and adjacent to microbial products (Figure 10 and Figure S11). There was no apparent change in the overall frequency of HAM56^+^ macrophages at the tips of the colonic crypts between SIV-uninfected, and acutely or chronically SIV-infected RMs (data not shown). The presence of high frequencies of macrophages without internalized microbial constituents, along with abundant extracellular microbial products suggested that GI tract macrophages in the later phase of acute infection and chronic stages of disease may become increasingly incapable of phagocytosing microbial products that translocate into the LP.

### Maintenance of the epithelial barrier and lack of microbial translocation in Sooty mangabeys

**Figure 11:**
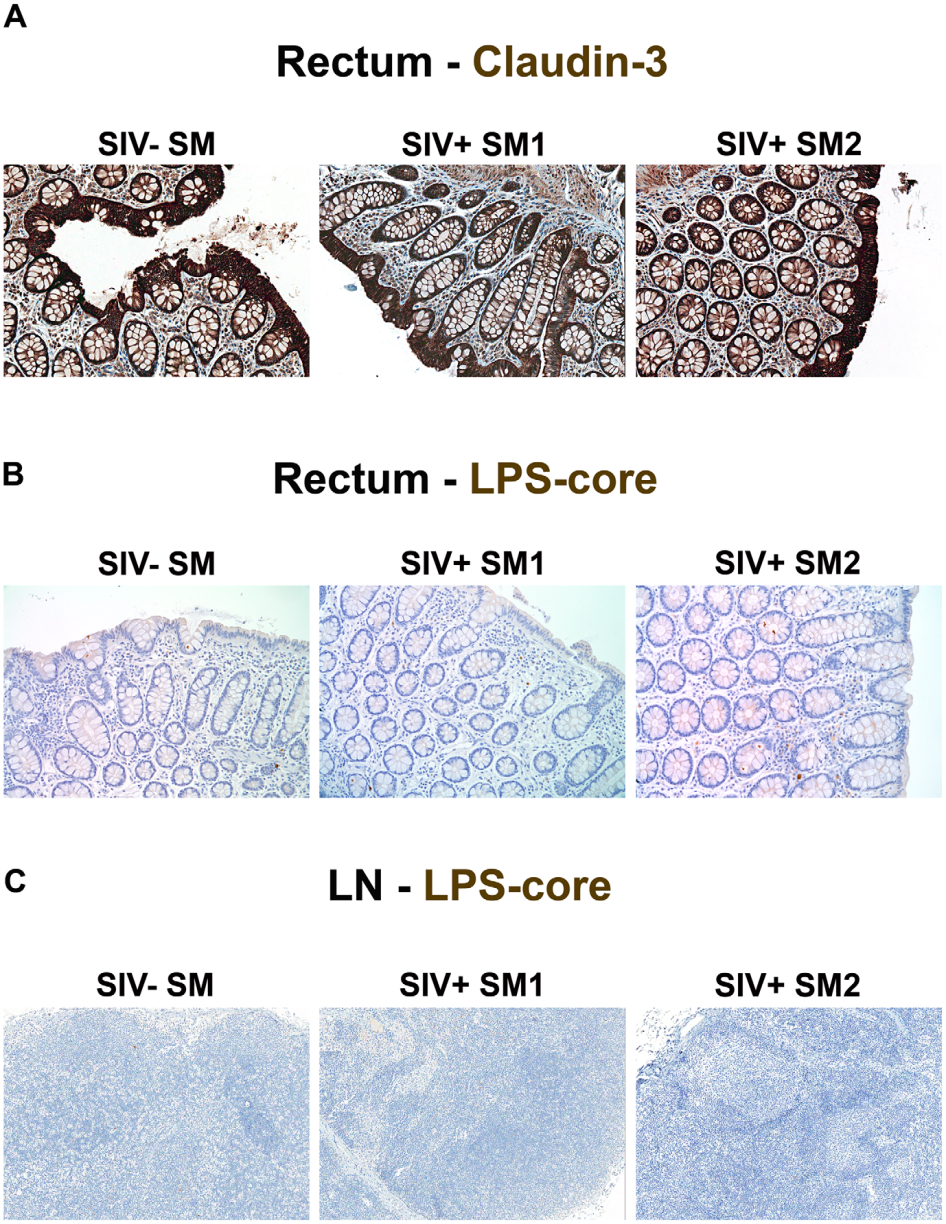
Absence of structural epithelial damage and microbial translocation in non-pathogenic infection of SMs. (**A**) Representative images (200×) of rectum stained for the tight junction protein claudin-3 (brown) show the complete maintenance of the epithelial barrier in SIVsmm-uninfected and SIVsmm-infected SMs. Representative images (200×) of (**B**) rectum and (**C**) peripheral lymph nodes (100×) stained for LPS-core antigen (brown) shows the absence of microbial translocation in SIVsmm-uninfected and SIVsmm-infected SMs.

A distinguishing feature of non-progressive infection in natural hosts of SIV is the absence of immune activation during the chronic phase of infection [6], [41], [42], [43], [44], [45]. Because elevated plasma LPS levels are absent in both chronically SIVsmm-infected SMs and SIVagm-infected AGMs [9], [45], [46], we evaluated the structural integrity of the intestinal epithelial barrier in chronically SIVsmm-infected SMs. In contrast to findings in chronically SIV-infected RMs, and consistent with the lack of LPS within the circulation of these SIV natural host animals [9], we found no evidence of damage to the integrity of the epithelial barrier (Figure 11A) and no infiltration of microbial products into the LP of large bowel (Figure 11B) or peripheral lymph nodes (Figure 11C). These data were consistent among 7 animals studied (n = 2, SIVsmm-uninfected SMs; n = 5, chronically SIVsmm-infected SMs; Table 1). Hence preservation of the tight epithelial barrier is associated with lack of microbial translocation and immune activation in non-progressive, natural SIV infection.

## Discussion

Indirect evidence has implicated microbial translocation from the gut as a factor contributing to pathological immune activation in chronic HIV/SIV infection, but direct evidence of translocation and demonstration of a plausible underlying mechanism have been lacking. Using an unbiased, comprehensive approach for quantitative and qualitative immunohistologic analysis of randomly selected tissue specimens obtained from non-human primates at various times relative to SIV infection, we have shown that: 1) microbial products can be found in the LP of the large bowel, in draining and distant lymph nodes, and in the liver of chronically SIV-infected RMs; 2) microbial translocation is associated with breakdown of the integrity of the epithelial barrier of SIV-infected RMs; 3) the extent of epithelial breakdown correlates with the extent of microbial translocation; 4) epithelial barrier breakdown and microbial translocation begin to be apparent during the late acute phase of infection (14 dpi); 5) the presence of microbial products in multiple anatomical sites is associated with expression of IFN-α and IL-18 in the absence of detectable local viral replication in the LP, consistent with direct induction of immune activation; 6) macrophages in chronically SIV-infected RMs appear dysfunctional with respect to their ability to phagocytose translocated microbial products; and 7) neither epithelial barrier breakdown nor infiltration of microbial products into the LP occur during the chronic phase of SIV infection of SMs.

We provide two lines of evidence linking microbial translocation to immune activation. First, we show that in pathogenic SIV infection of RMs, damage to the integrity of the epithelial barrier of the GI tract is associated with microbial translocation, and that microbial translocation is linked to local immune activation, based on co-localization of microbial products and production of the immunoinflammatory cytokines IFNα and IL-18, including in lymph nodes anatomically distant from the GI tract. Second, in marked contrast, in SIV-infected SMs where immune activation is quickly resolved in the acute stage of infection [47], and chronic infection is not accompanied by persistent immune activation, we found neither damage to the intestinal barrier nor microbial translocation.

Recent *in vitro* studies with peripheral blood lymphocytes from SMs have been interpreted to suggest that a lack of type I IFN cytokine response to SIV RNA accounts for the typically nonprogressive nature of SIV infection in SMs [48]. Furthermore, these data have been used to suggest that the raised plasma LPS levels observed in chronically SIV-infected RMs and HIV-infected humans, are simply markers of damage to the GI tract and that the microbial translocation that is reflected in elevated plasma LPS levels does not contribute significantly to causing systemic immune activation [48]. However not only were LPS levels increased in chronically-infected individuals, but sCD14 and LPS binding protein levels were also increased [9], [11], [13]. These data strongly suggested that LPS was directly stimulating the immune system *in vivo*. In the present study, we show directly that damage to the integrity of the epithelial barrier of the GI tract allows microbial products to infiltrate into the LP and this infiltration is associated with local immune activation demonstrated by co-localization of microbial products within the LP with IFNα and IL-18 in MesLN. As only limited viral replication is demonstrable in the LP of the GI tract of chronically SIV-infected RMs, the damage to the GI epithelial lining, microbial translocation and local immune activation are unlikely to be caused by the direct effects of local viral replication. Rather, where rare infected cells are seen in the LP underlying damaged mucosa, it is more likely that the chronic immune activation, due to translocated microbial products, has provided activated CD4^+^ T cell targets for the virus.

Taken together, these data suggest that microbial translocation, resulting from damage to the GI tract epithelial barrier and impaired macrophage-mediated phagocytosis, results in immune activation during the chronic phase of HIV/SIV infection of humans and RMs. Importantly, we found a significant correlation between the extent of damage to the epithelial barrier of the colon and the amount of LPS within the underlying mucosa and the extent of translocated microbial products in draining and remote lymph node tissues. The extent of microbial constituents present in lymph node tissues correlated with the extent of local evidence of immune activation, further substantiating the link. Moreover, while we find that microbial translocation begins during the acute phase of infection, our previous work had indicated that elevated levels of microbial products were not seen in plasma until the chronic phase [9]. Our data suggest that microbial products are localized within tissue macrophages during the acute phase thus limiting their circulation.

The causes of damage to the integrity of the epithelial barrier of the GI tract are likely to be multifaceted, but in the chronic stages of SIV infection seem unlikely to be due to direct virotoxic effects, given the lack of association with very low levels of demonstrable local viral replication in the LP relative to the extensive epithelial damage. One possible mechanism may be related to the preferential loss of Th17 cells in the GI tract in progressive immunodeficiency lentiviral infections [25], [26], because Th17 cells produce cytokines important for enterocyte proliferation and antibacterial defensins [49], [50], [51] and IL-17 has recently been shown to suppress Th1-mediated damage to gut epithelium. Importantly, preservation of this T cell subset in the gut of chronically SIVsmm-infected SMs and SIVagm-infected AGMs [26], [52] and the sparing of the epithelial barrier of SIVsmm infected SMs we show here supports this mechanism.

We speculate that the association we found between immune activation, microbial translocation and chronic stages of SIV infection, and similarly, later stages of HIV-1 infection, reflects damage to the structural integrity of the GI tract and a potential “deficiency” of the GI tract macrophage-phagocytic system. Our observation that intestinal macrophages from SIV-infected RMs, which are generally not proinflammatory [40], are unable to clear translocated microbial products, within the LP, and could lead to increased proinflammatory responses locally are supported by several groups findings that showed: i) impaired monocyte phagocytosis in HIV-infected individuals [53]; ii) reduced LPS-mediated enhancement of phagocytosis in monocytes HIV-infected individuals compared to healthy donors [54]; and iii) significantly higher colonic mucosa proinflammatory mRNA expression levels (e.g. TNF-α, IFN-γ, and IL-6) in HIV-infected patients than in control patients [55]. These data certainly warrant further investigation into the functional properties of tissue macrophages from HIV/SIV-infected individuals and the mechanisms underlying their apparent dysfunction. While increased microbial translocation begins in the late acute stage of SIV infection, it was not until the later stages of infection that the capacity of macrophages for clearance was apparently affected, suggesting that microbial translocation has an increasing major contribution to immune activation as the host progresses towards disease. The evidence for this model comes from images of MesLN and AxLN stained for bacterial products in the chronic stage that showed dramatically increased extracellular bacterial constituents in the late AIDS stage of SIV infection, versus the mainly cellular staining of LPS at earlier stages. Taken together, these data strongly suggest that in SIV infection of RMs, and by extension, HIV infection of humans, damage to the epithelial barrier of the GI tract leads to levels of microbial translocation that exceed the capacity of host defense mechanisms to sequester away microbial constituents from secondary lymphatic tissues, resulting in persistent immune activation that contributes importantly to pathogenesis during the chronic phase of infection. Understanding the factors underlying damage to the integrity of the epithelial barrier and macrophage deficiencies that we report may lead to novel therapeutic interventions that aim to reduce microbial translocation and the deleterious effects of the consequent immune activation.

## Materials and Methods

### Animals and tissues

To characterize the extent of microbial translocation in the gastrointestinal tract in SIV infection, we studied tissues from an assembled cohort of SIV-uninfected and infected RMs and SIVsmm-infected and uninfected SMs originally involved in separate studies (summarized in Table 1). Tissues (colon and LNs) were obtained at necropsy from 13 rhesus macaques (*Macaca mulatta*) of Indian origin euthanized 1–28 days after atraumatic intravaginal infection with SIVmac251 or SIVmac239 as described elsewhere [56]. Six additional SIV-negative RMs were used as controls. In a separate study, tissues were obtained at necropsy from 13 adult RMs chronically infected with SIVmac239 or SIVsmE660 that were sacrificed either because of end-stage disease (AIDS; defined by opportunistic infections, lymphomas, or a diagnosis of wasting, based on greater than 15% body mass weight loss, n = 5) or protocol specified experimental end point (Non-AIDS; n = 8). For immunohistochemistry studies, samples were very quickly processed into fixative to avoid potential artifacts associated with post-mortem tissue changes. The post mortem interval, the time from euthanasia until collection of lymph nodes and GI tract segments were placed into fixative, ranged from ∼10–30 minutes and was consistent over four independent primate facilities contributing tissues to the present study. The GI tract segments sampled at necropsy were representative and were not selected with regard to any visually apparent lesions or other pathology. In a third study, LNs and rectal biopsies were obtained from 2 SIVsmm-negative SMs (*Cercocebus atys*) and 5 SMs that were naturally infected with SIVsmm as previously described [47]. Animals were housed and cared in accordance with American Association for Accreditation of Laboratory Animal Care standards in AAALAC accredited facilties, and all animal procedures were performed according to protocols approved by the Institutional Animal Care and Use Committees of the National Cancer Institute, California National Primate Research Center or Yerkes National Primate Research Center (Table 1). Unfortunately, paraffin-embedded tissue from colon, mesLN, and axLN samples were not available from all animals.

### Plasma viral loads

Plasma samples were analyzed for SIV vRNA by using a quantitative branched DNA (bDNA) assay [53] or using a fluorescent resonance energy transfer probe-based real-time RT-PCR (TaqMan) assay that provides a threshold sensitivity of 125 copy Eq/ml, as previously described [57]. All PCR reactions were run on ABI Prism 7700 Sequence Detection System and the fluorescent signal-based quantitation of viral RNA copy numbers in test samples was determined by ABI sequence detection software (Applied Biosystems, Foster City, CA).

### Immunohistochemistry, ISH and FISH

Immunohistochemical staining and SIV *in situ* hybridization were performed as previously described [58]. In brief, unselected specimens of tissues of interest were obtained at necropsy, fixed, and paraffin embedded. Immunohistochemistry was performed using a biotin-free polymer approach (MACH-3; Biocare Medical) on 5-µm tissue sections mounted on glass slides, which were dewaxed and rehydrated with double-distilled water. Antigen retrieval was performed by heating sections in 1× DIVA Decloaker reagent (Biocare Medical) in a pressure cooker (Biocare Medical) followed by cooling to room temperature. All slides were stained using the intelliPATH FLX autostaining system (Biocare Medical) according to experimentally determined optimal conditions. This included blocking tissues with Blocking Reagent (Biocare Medical) for 10 min followed by an additional blocking step with TNB (0.1 M Tris-HCL (pH 7.5), 0.15 M NaCl, and 0.5% Blocking Reagent (NEN)) containing 2% Blocking Reagent and 100 µg/mL goat ChromePure IgG (Jackson Immunoresearch) for 10 minutes at room temperature. Endogenous peroxidase was blocked with 1.5% (v/v) H_2_O_2_ in TBS (pH 7.4). Primary antibodies were diluted in TNB containing 2% Blocking Reagent and 100 µg/mL goat ChromePure IgG for 1 h at room temperature. Mouse or rabbit MACH-3 secondary polymer systems (Biocare Medical) were applied for 20 minutes each. Double immunohistochemical staining was performed on colon and lymph node sections with either mouse monoclonal anti-LPS-core and rabbit polyclonal anti-IL18 antibodies or mouse monoclonal anti-IFNα and rabbit polyclonal anti-*E.coli* using the MACH-2 multiplex staining system (Biocare Medical) according to manufacturer's instructions. Sections were developed with ImmPACT DAB (Vector Laboratories) and/or Vulcan Fast Red chromogen (Biocare Medical), counterstained with hematoxylin, and mounted in Permount (Fisher Scientific).

All stained slides were scanned at high magnification (400×) using the ScanScope CS System (Aperio Technologies, Inc.) yielding high-resolution data from the entire tissue section. Bacterial PNA FISH was performed using the universal bacterial 16 s ribosomal RNA specific (UniBac) FITC conjugated PNA probe (AdvanDx, Inc.) according to manufacturer's instructions, with the exception that a heat induced epitope retrieval pretreatment step was performed in 1× Diva retrieval buffer (Biocare Medical) for 20 minutes in a 95°C water bath prior to hybridization. FISH samples were examined and imaged using a Nikon 80i upright fluorescent microscope (Nikon Instruments, Inc) equipped with a BrightLine multiband bandpass FITC/Texas Red filter (Semrock; data not shown). Primary antibodies used were: mouse anti-human Interferon-α (clone MMHA-2; PBL InterferonSource), mouse anti-LPS core (clone WN1 222-5; Hycult or provided by Dr. Robin Barclay), mouse anti-macrophage (clone HAM56; Dako), mouse anti-cytokeratin (clone MNF116; Dako), polyclonal rabbit anti-*E. coli* (Dako), polyclonal rabbit anti-IL-18 (Sigma Prestige Antibodies by Atlas Antibodies) and polyclonal rabbit anti-Claudin-3 (Labvision).

### Confocal fluorescent microscopy

Immunofluorescent confocal microscopy was performed on treated slides as above, but stained overnight with primary antibodies at 4°C, washed, stained with fluorescently conjugated secondary antibodies for 1 h in the dark, counterstained with DAPI (Molecular Probes), mounted in AquaPoly mount (Polysciences, Inc.) and imaged using a Olympus FluoView FV1000. Z-stack images were taken for each high power field that spanned the entire 5 µm tissue section and representative 3-D projections from z-stack images were generated using Imaris 7.0.0 software (Bitplane Inc.). Secondary antibodies used were donkey anti-mouse IgG Alexa Fluor 488 and donkey anti-rabbit IgG Alexa Fluor 555 (all from Molecular Probes).

### Quantitative image analysis

To quantify microbial translocation (LPS) into the LP and the extent of epithelial barrier damage (claudin-3), 5-µm thick sections were cut from paraffin blocks of unselected tissue sections obtained at necropsy and stained with either monoclonal antibody against LPS-core or polyclonal antibody for claudin-3 and counter stained with heamotoxalin. High power (400×) whole tissue scans were obtained using an Aperio ScanScope as described above and imported into Photoshop CS3 (Adobe Systems Inc., Mountain View, California, USA). Images were manually trimmed to remove the submucosae, muscularis and residual luminal content, leaving only the LP mucosae to analyze. The percent area of the LP staining for LPS was determined essentially as previously described using Photoshop CS3 tools with plug-ins from Reindeer Graphics [39], [47]. The percent area of LN staining for LPS was determined from whole LN scans as above but without the need to trim the image. The proportion of the epithelial barrier that was damaged during SIV infection was first determined by manually tracing (in red) the area of the lumen/GI epithelial tract interface that had no claudin-3 staining epithelial cells, using the brush tool in Photoshop CS3. The remaining claudin-3 staining intact epithelial cell regions were then manually traced (in black). The percent damage was calculated by determining the proportion of the image that was red (lack of claudin-3 stain) compared to the total epithelial surface area (red+black) using plug-in tools from Reindeer Graphics.

### Statistical tests

Spearman's rank correlation and Mann-Whitney tests were performed using Prism 4.0 software (Prism, San Diego, CA).

## Supporting Information

Figure S1 Identification of microbial translocation (LPS) in large bowel of chronically SIV^+^ RMs. Low magnification whole tissue (top panel), 40× (middle panel) and 100× (bottom panel) images from high power whole tissue scans of colon immunohistochemically stained for LPS-core antigen (brown). Rectangles represent regions of the colon magnified in the successive images, while the rectangles displayed in the 100× lower panel images represent the region magnified and displayed in Figure 1. Scale bars  = 1 mm.
(4.38 MB TIF)

Figure S2 Identification of microbial translocation (*E. coli*) in large bowel of chronically SIV^+^ RMs. Low magnification whole tissue (top panel), 40× (middle panel) and 100× (bottom panel) images from high power whole tissue scans of colon immunohistochemically stained with a polyclonal antibody against *E. coli* (brown). Rectangles represent regions of the colon magnified in the successive images, while the rectangles displayed in the 100× lower panel images represent the region magnified and displayed in Figure 2. Scale bars  = 1 mm.
(4.42 MB TIF)

Figure S3 Identification of microbial translocation in gut draining MesLN of chronically SIV^+^ RMs. Low magnification whole tissue (top panel), 40× (middle panel) and 100× (bottom panel) images from high power whole tissue scans of MesLN immunohistochemically stained for LPS-core antigen (brown). Rectangles represent regions of the colon magnified in the successive images, while the rectangles displayed in the 100× lower panel images represent the region magnified and displayed in Figure 3. Scale bars  = 1 mm.
(5.35 MB TIF)

Figure S4 Identification of microbial translocation in systemic distal AxLN of chronically SIV^+^ RMs. Low magnification whole tissue (top panel), 40× (middle panel) and 100× (bottom panel) images from high power whole tissue scans of AxLN immunohistochemically stained for LPS-core antigen (brown). Rectangles represent regions of the colon magnified in the successive images, while the rectangles displayed in the 100× lower panel images represent the region magnified and displayed in Figure 4. Scale bars  = 1 mm.
(5.24 MB TIF)

Figure S5 Identification of microbial translocation (*E. coli*) in the liver of chronically SIV^+^ RMs. Low magnification whole tissue (top panel), 40× (middle panel) and 100× (bottom panel) images from high power whole tissue scans of liver immunohistochemically stained with a polyclonal antibody against *E. coli* (brown). Rectangles represent regions of the colon magnified in the successive images, while the rectangles displayed in the 100× lower panel images represent the region magnified and displayed in Figure 5. In 40× and 100× images, arrows point to portal triads, while arrow heads point to central veins. Scale bars  = 1 mm.
(4.89 MB TIF)

Figure S6 Spatial localization of microbial products with type I IFNα^+^ cells in colon. Images (200×) of colon stained for both *E. coli* (brown) and IFNα (red) from uninfected and chronically SIV-infected Non-AIDS and AIDS RMs.
(8.30 MB TIF)

Figure S7 Spatial localization of microbial products with an effector marker of immune activation, IL-18, in MesLN. Images (400×) of MesLN stained for both LPS (brown) and IL-18 (red) from uninfected and SIV-infected Non-AIDS RMs. Note the spatial proximity of LPS^+^ cells and extracellular LPS with IL-18^+^ cells in MesLN. Scale bars  = 50 µm.
(10.20 MB TIF)

Figure S8 Damage to the integrity of the epithelial barrier in chronic SIV^+^ RMs. Low magnification whole tissue (top panel), 40× (middle panel) and 100× (bottom panel) images from high power whole tissue scans of colon from SIV uninfected and chronically SIV^+^ RMs immunohistochemically stained for the tight junction protein claudin-3 (brown). Rectangles represent regions of the colon magnified in the successive images, while the rectangles displayed in the 100× lower panel images represent the region magnified and displayed in Figure 7. Scale bars  = 1 mm.
(4.68 MB TIF)

Figure S9 GI tract damage results in PMN infiltration within the colon of chronically SIV^+^ RM. (A) Representative images (200×) of the colon stained for myeloperoxidase (brown) as a marker for PMNs. Note the increasing accumulation of myeloperoxidase+ PMNs adjacent to epithelial lesions in chronically SIV^+^ RM, reflecting a tissue response to loss of epithelial integrity, but the lack of this association seen in early acute SIV^+^ RM (4 dpi). (B) Random high power 400× images (10–15) of gut LP were taken and the percent area staining for myeloperoxidase (PMN) were determined in early/acute and chronic SIV^+^ RM.
(3.11 MB TIF)

Figure S10 Damage to the integrity of the epithelial barrier is associated with increased enterocyte proliferation. Low magnification whole tissue (top panel), 40× (middle panel) and 100× (bottom panel) images from high power whole tissue scans of colon from SIV uninfected and chronically SIV^+^ RMs immunohistochemically stained for Ki67 (brown). Rectangles represent regions of the colon magnified in the successive images, while the rectangles displayed in the 100× lower panel images represent the region magnified and displayed in Figure 9. Scale bars  = 1 mm.
(4.66 MB TIF)

Figure S11 Quantitative image analysis of the frequency of HAM56^+^ macrophages that are *E. coli* ^+^ compared to the proportion of HAM56^−^ *E.coli* ^+^ events in SIV-uninfected and acutely and chronically SIV-infected RMs. Note that throughout the acute phase of infection (1–21 dpi), most microbial products are within or associated with macrophages, however, starting at 28 dpi and extending into the chronic stage macrophages, although still abundant in the GI tract, become progressively inefficient/dysfunction in their ability to bind and/or phagocytose microbial products that cross the epithelial barrier.
(0.13 MB TIF)
