# Supplementary figures and images for: Damaged Intestinal Epithelial Integrity Linked to Microbial Translocation in Pathogenic Simian Immunodeficiency Virus Infections
Source: PLoS Pathog. 2010 Aug 19;6(8):e1001052. doi: 10.1371/journal.ppat.1001052 (PMC2924359; doi:10.1371/journal.ppat.1001052)

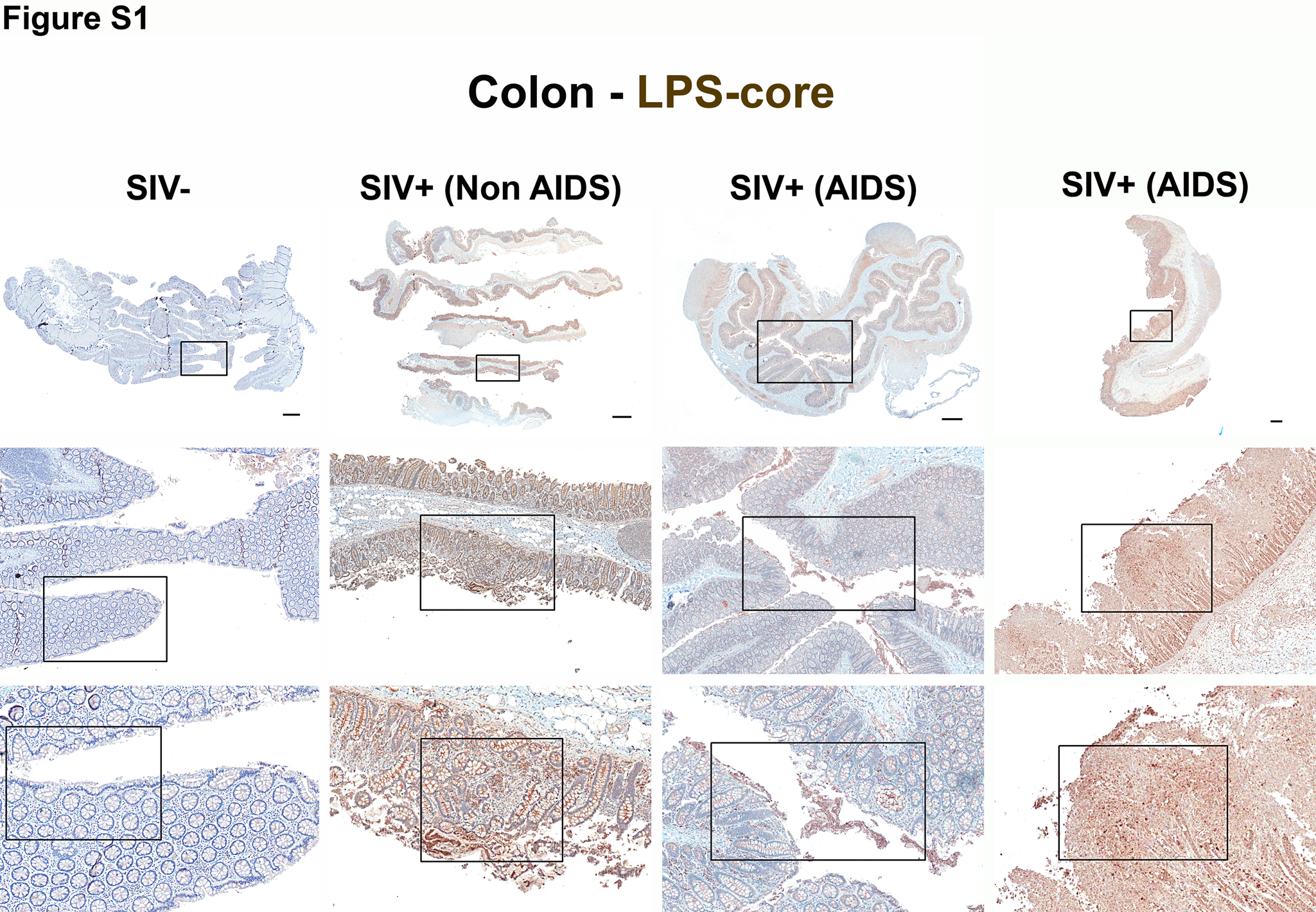

Supplement: Figure S1 — Identification of microbial translocation (LPS) in large bowel of chronically SIV+ RMs. Low magnification whole tissue (top panel), 40× (middle panel) and 100× (bottom panel) images from high power whole tissue scans of colon immunohistochemically stained for LPS-core antigen (brown). Rectangles represent regions of the colon magnified in the successive images, while the rectangles displayed in the 100× lower panel images represent the region magnified and displayed in Figure 1. Scale bars = 1 mm. (4.38 MB TIF) [file ppat.1001052.s001.tif]

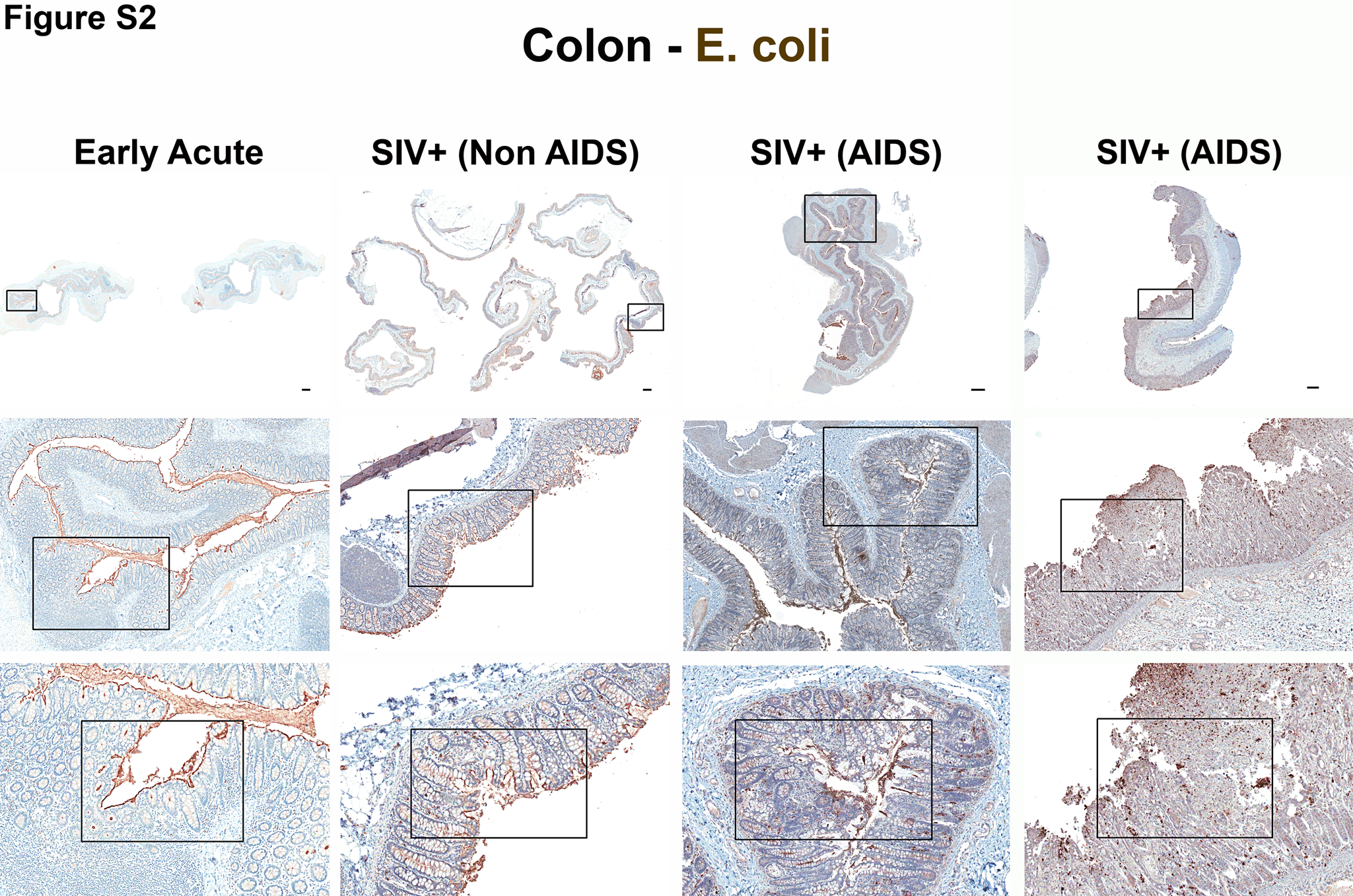

Supplement: Figure S2 — Identification of microbial translocation (E. coli) in large bowel of chronically SIV+ RMs. Low magnification whole tissue (top panel), 40× (middle panel) and 100× (bottom panel) images from high power whole tissue scans of colon immunohistochemically stained with a polyclonal antibody against E. coli (brown). Rectangles represent regions of the colon magnified in the successive images, while the rectangles displayed in the 100× lower panel images represent the region magnified and displayed in Figure 2. Scale bars = 1 mm. (4.42 MB TIF) [file ppat.1001052.s002.tif]

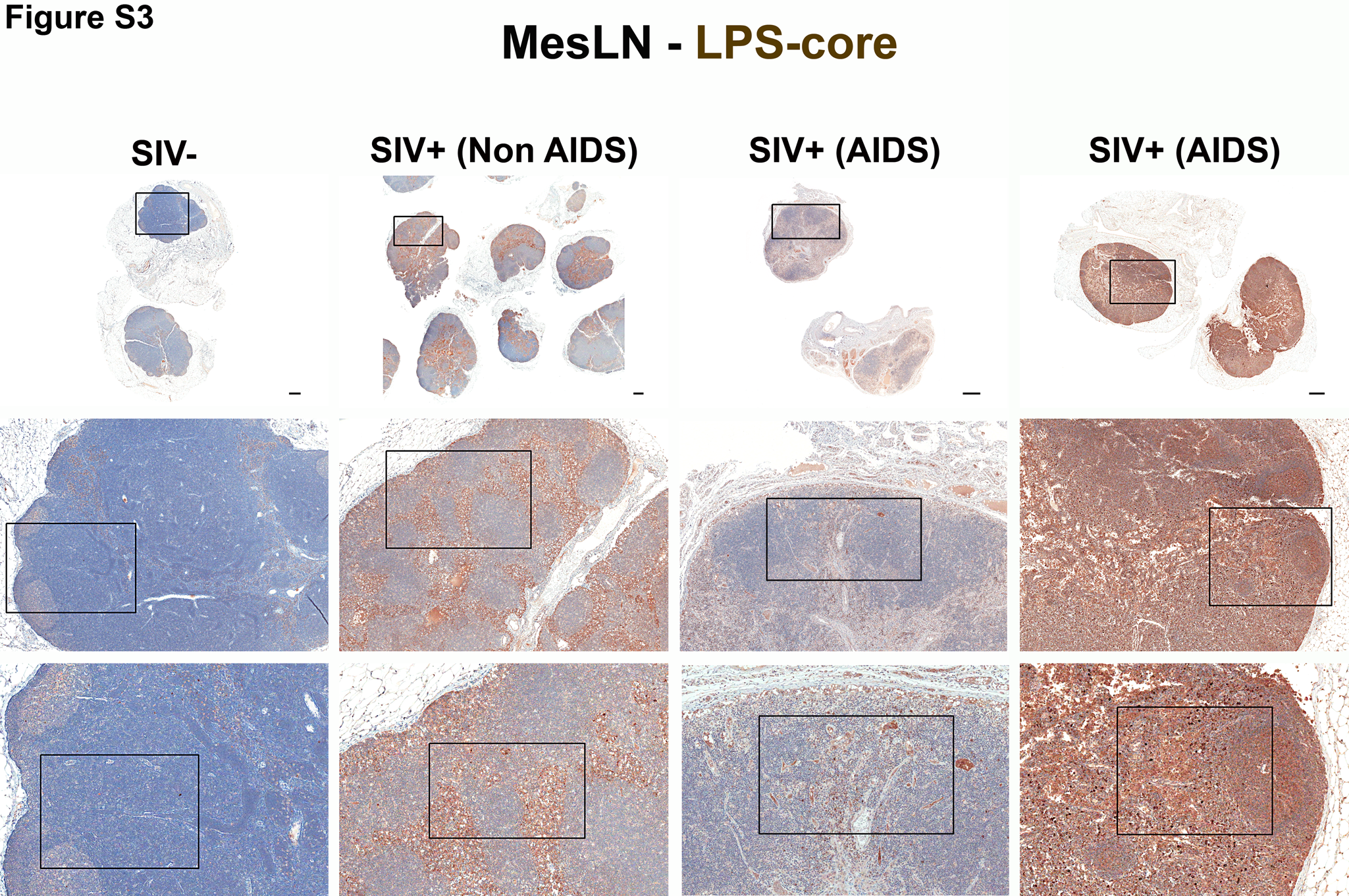

Supplement: Figure S3 — Identification of microbial translocation in gut draining MesLN of chronically SIV+ RMs. Low magnification whole tissue (top panel), 40× (middle panel) and 100× (bottom panel) images from high power whole tissue scans of MesLN immunohistochemically stained for LPS-core antigen (brown). Rectangles represent regions of the colon magnified in the successive images, while the rectangles displayed in the 100× lower panel images represent the region magnified and displayed in Figure 3. Scale bars = 1 mm. (5.35 MB TIF) [file ppat.1001052.s003.tif]

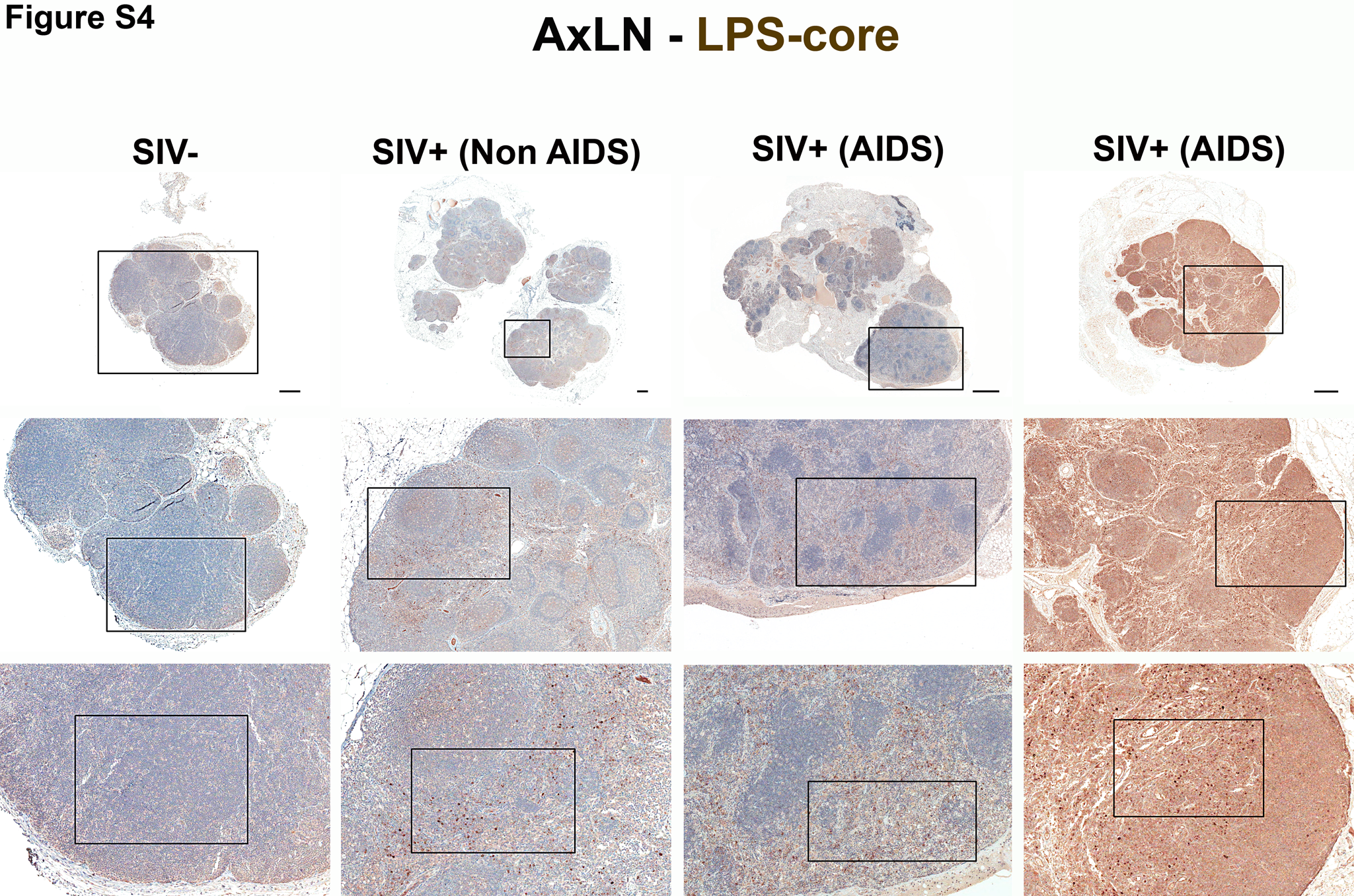

Supplement: Figure S4 — Identification of microbial translocation in systemic distal AxLN of chronically SIV+ RMs. Low magnification whole tissue (top panel), 40× (middle panel) and 100× (bottom panel) images from high power whole tissue scans of AxLN immunohistochemically stained for LPS-core antigen (brown). Rectangles represent regions of the colon magnified in the successive images, while the rectangles displayed in the 100× lower panel images represent the region magnified and displayed in Figure 4. Scale bars = 1 mm. (5.24 MB TIF) [file ppat.1001052.s004.tif]

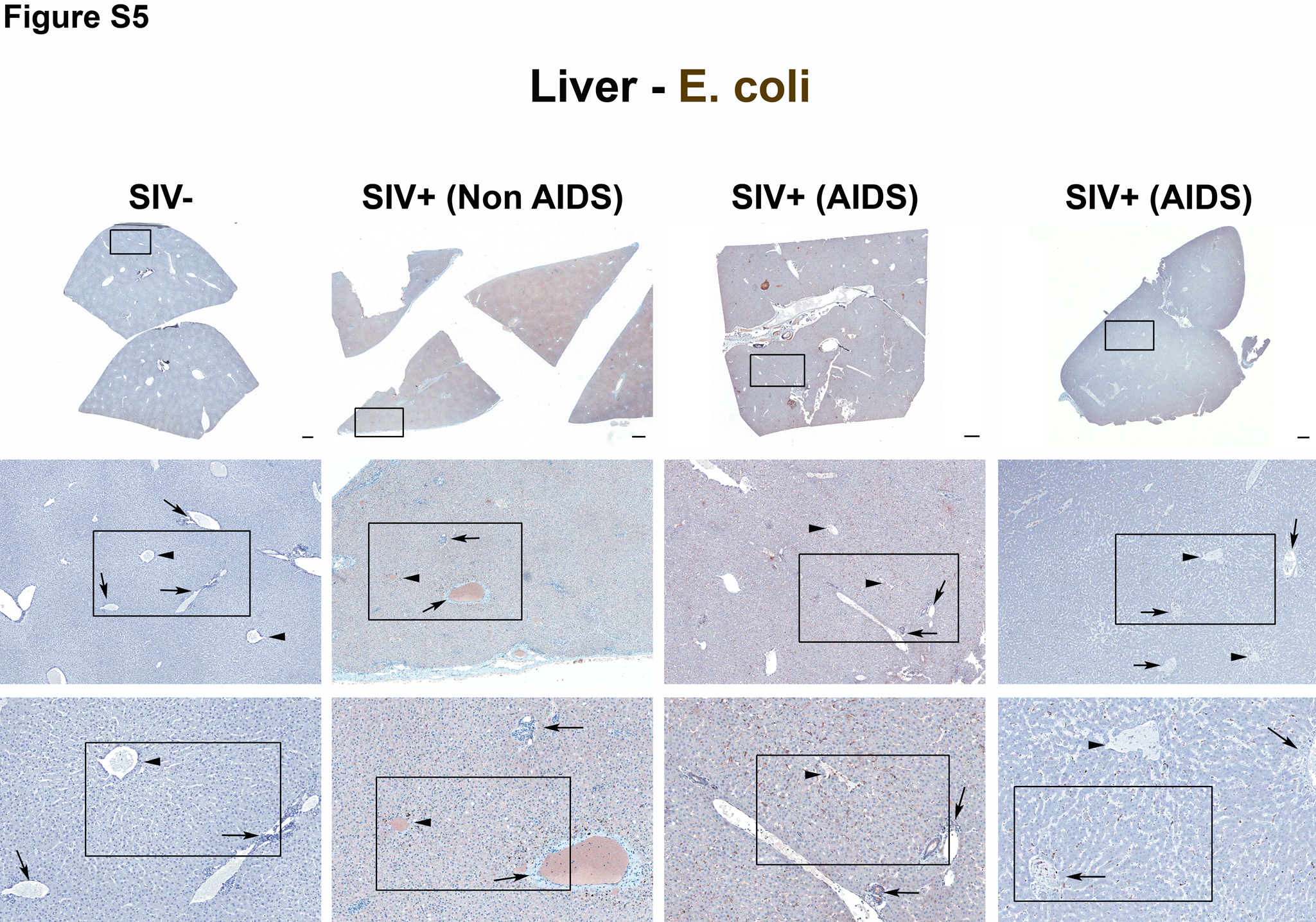

Supplement: Figure S5 — Identification of microbial translocation (E. coli) in the liver of chronically SIV+ RMs. Low magnification whole tissue (top panel), 40× (middle panel) and 100× (bottom panel) images from high power whole tissue scans of liver immunohistochemically stained with a polyclonal antibody against E. coli (brown). Rectangles represent regions of the colon magnified in the successive images, while the rectangles displayed in the 100× lower panel images represent the region magnified and displayed in Figure 5. In 40× and 100× images, arrows point to portal triads, while arrow heads point to central veins. Scale bars = 1 mm. (4.89 MB TIF) [file ppat.1001052.s005.tif]

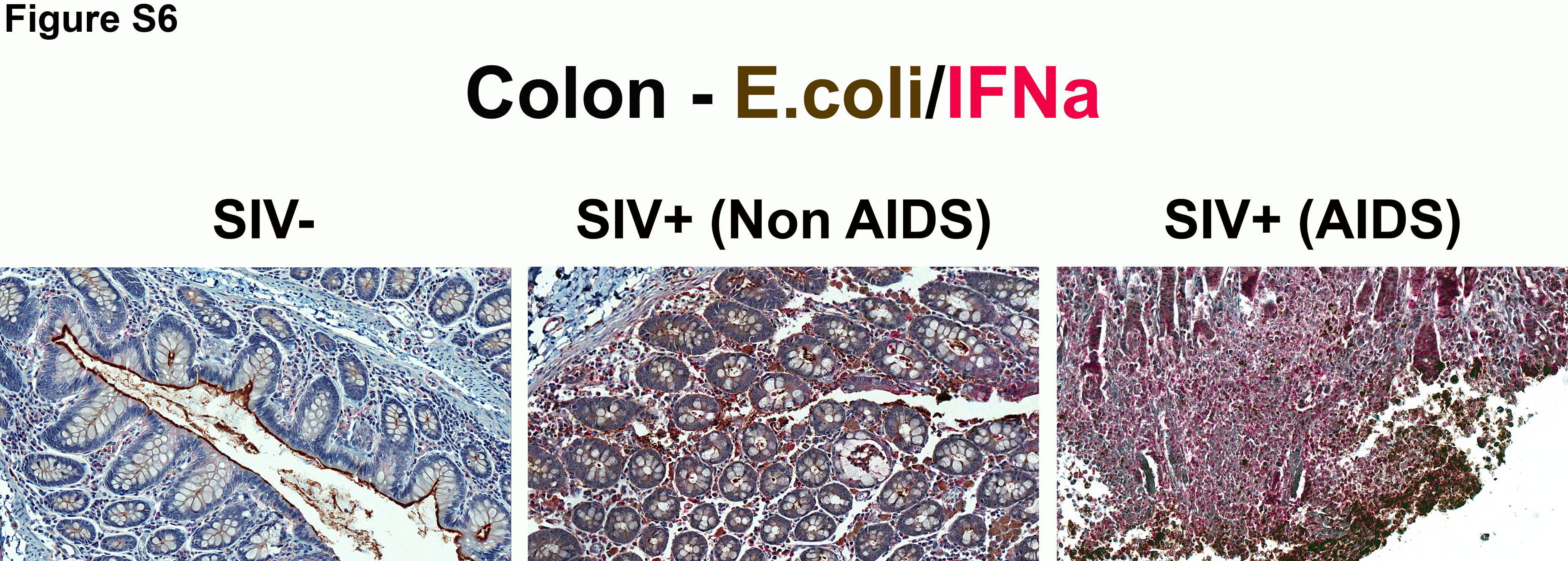

Supplement: Figure S6 — Spatial localization of microbial products with type I IFNα+ cells in colon. Images (200×) of colon stained for both E. coli (brown) and IFNα (red) from uninfected and chronically SIV-infected Non-AIDS and AIDS RMs. (8.30 MB TIF) [file ppat.1001052.s006.tif]

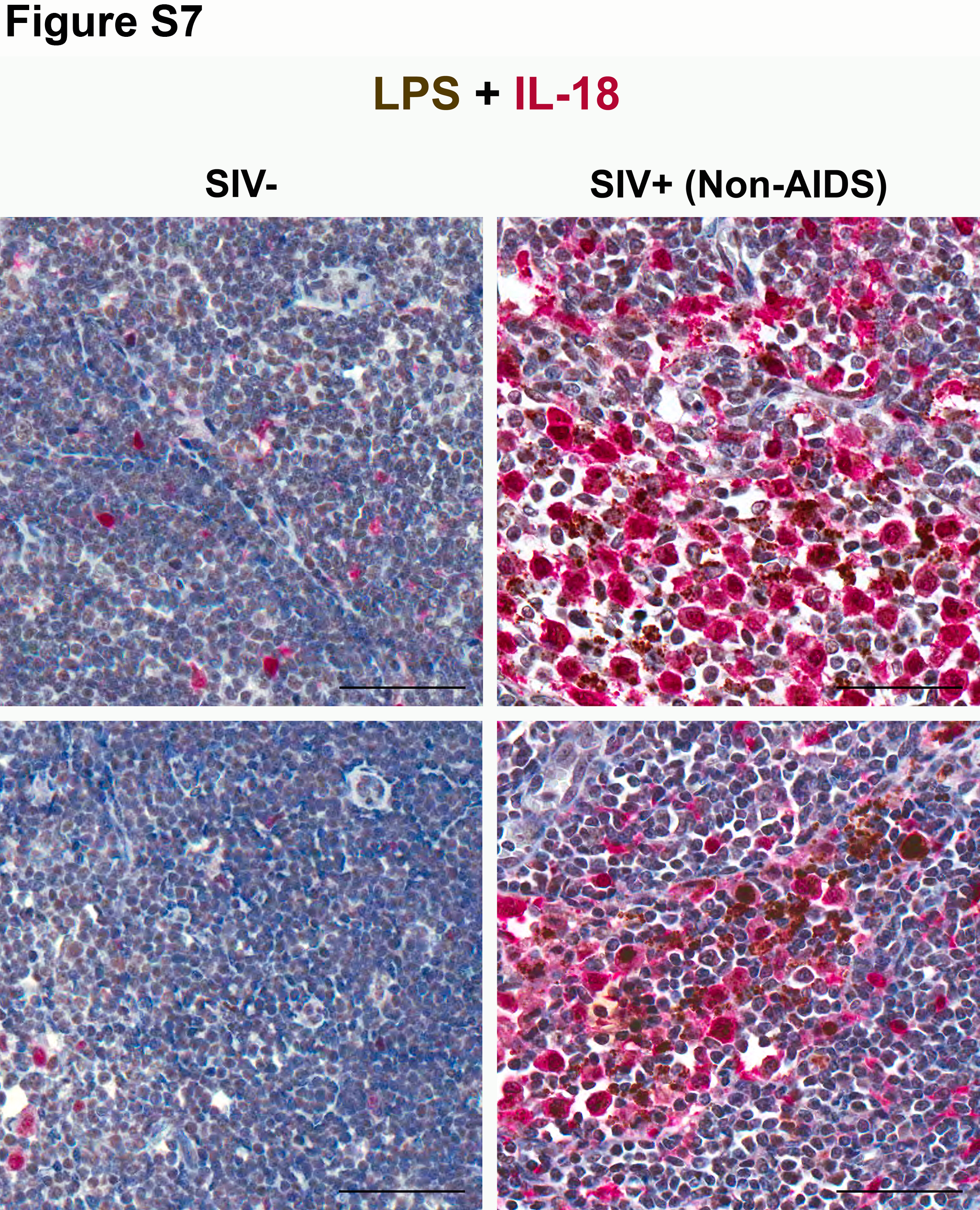

Supplement: Figure S7 — Spatial localization of microbial products with an effector marker of immune activation, IL-18, in MesLN. Images (400×) of MesLN stained for both LPS (brown) and IL-18 (red) from uninfected and SIV-infected Non-AIDS RMs. Note the spatial proximity of LPS+ cells and extracellular LPS with IL-18+ cells in MesLN. Scale bars = 50 µm. (10.20 MB TIF) [file ppat.1001052.s007.tif]

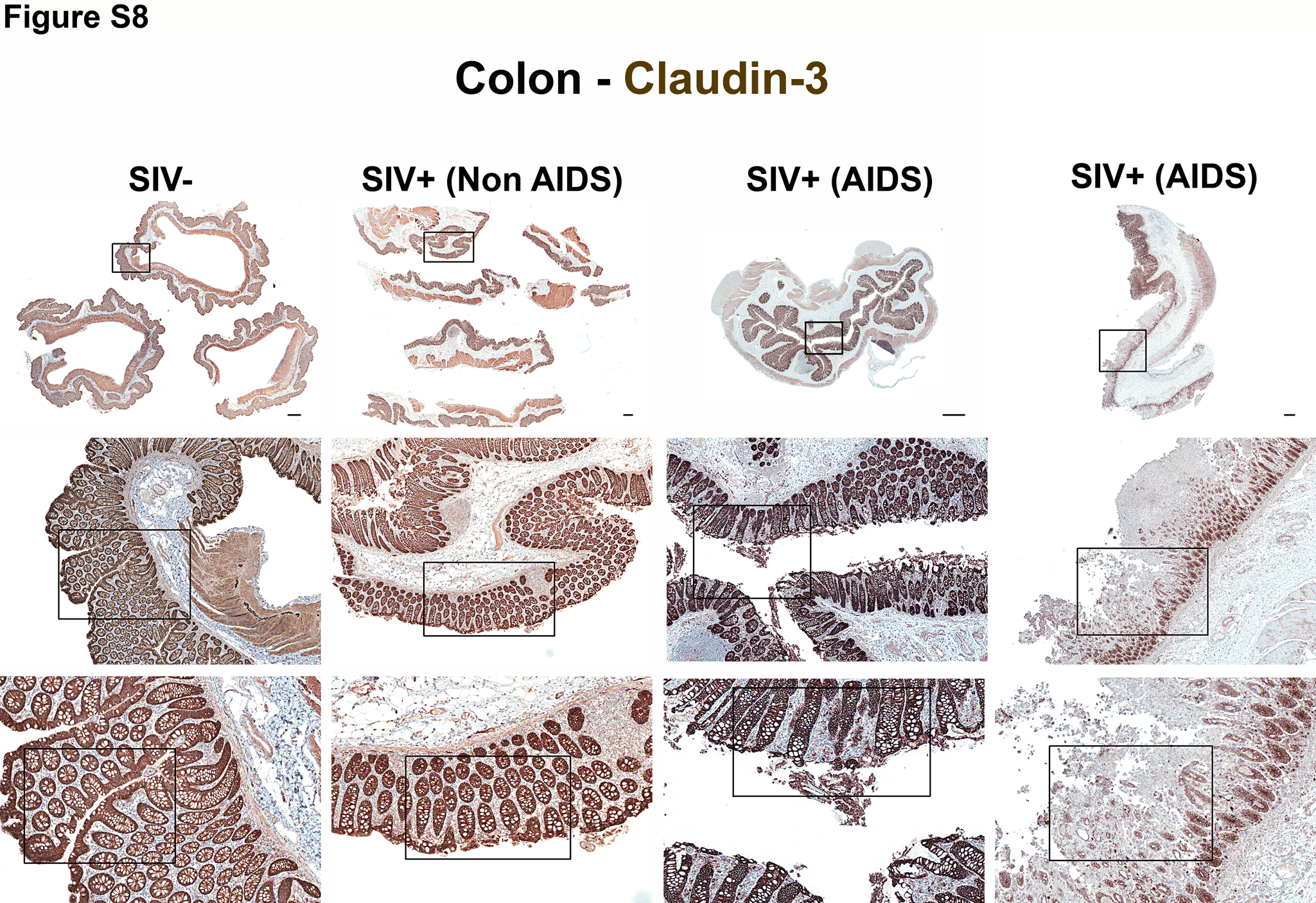

Supplement: Figure S8 — Damage to the integrity of the epithelial barrier in chronic SIV+ RMs. Low magnification whole tissue (top panel), 40× (middle panel) and 100× (bottom panel) images from high power whole tissue scans of colon from SIV uninfected and chronically SIV+ RMs immunohistochemically stained for the tight junction protein claudin-3 (brown). Rectangles represent regions of the colon magnified in the successive images, while the rectangles displayed in the 100× lower panel images represent the region magnified and displayed in Figure 7. Scale bars = 1 mm. (4.68 MB TIF) [file ppat.1001052.s008.tif]

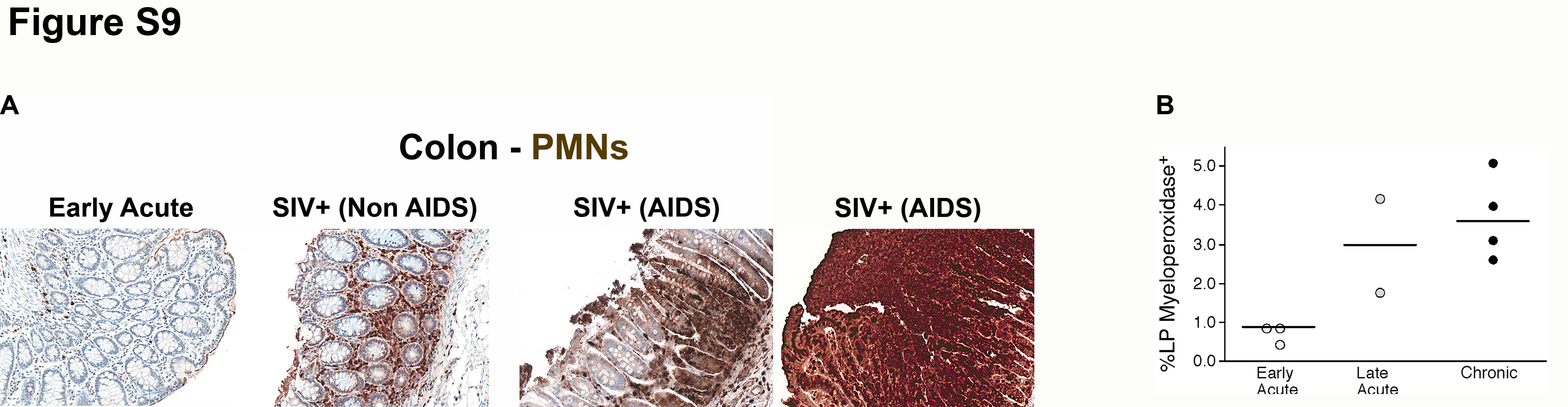

Supplement: Figure S9 — GI tract damage results in PMN infiltration within the colon of chronically SIV+ RM. (A) Representative images (200×) of the colon stained for myeloperoxidase (brown) as a marker for PMNs. Note the increasing accumulation of myeloperoxidase+ PMNs adjacent to epithelial lesions in chronically SIV+ RM, reflecting a tissue response to loss of epithelial integrity, but the lack of this association seen in early acute SIV+ RM (4 dpi). (B) Random high power 400× images (10–15) of gut LP were taken and the percent area staining for myeloperoxidase (PMN) were determined in early/acute and chronic SIV+ RM. (3.11 MB TIF) [file ppat.1001052.s009.tif]

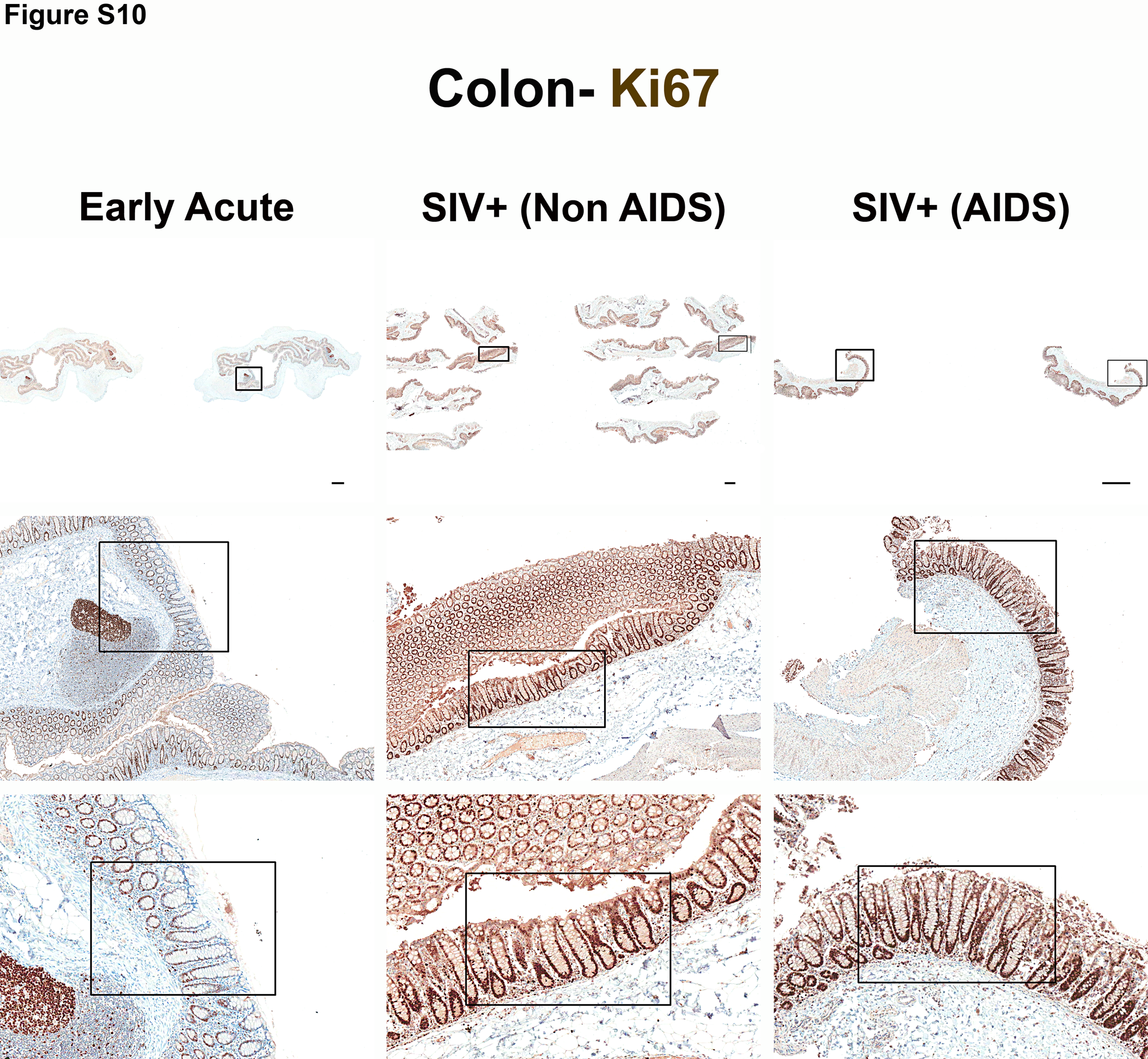

Supplement: Figure S10 — Damage to the integrity of the epithelial barrier is associated with increased enterocyte proliferation. Low magnification whole tissue (top panel), 40× (middle panel) and 100× (bottom panel) images from high power whole tissue scans of colon from SIV uninfected and chronically SIV+ RMs immunohistochemically stained for Ki67 (brown). Rectangles represent regions of the colon magnified in the successive images, while the rectangles displayed in the 100× lower panel images represent the region magnified and displayed in Figure 9. Scale bars = 1 mm. (4.66 MB TIF) [file ppat.1001052.s010.tif]

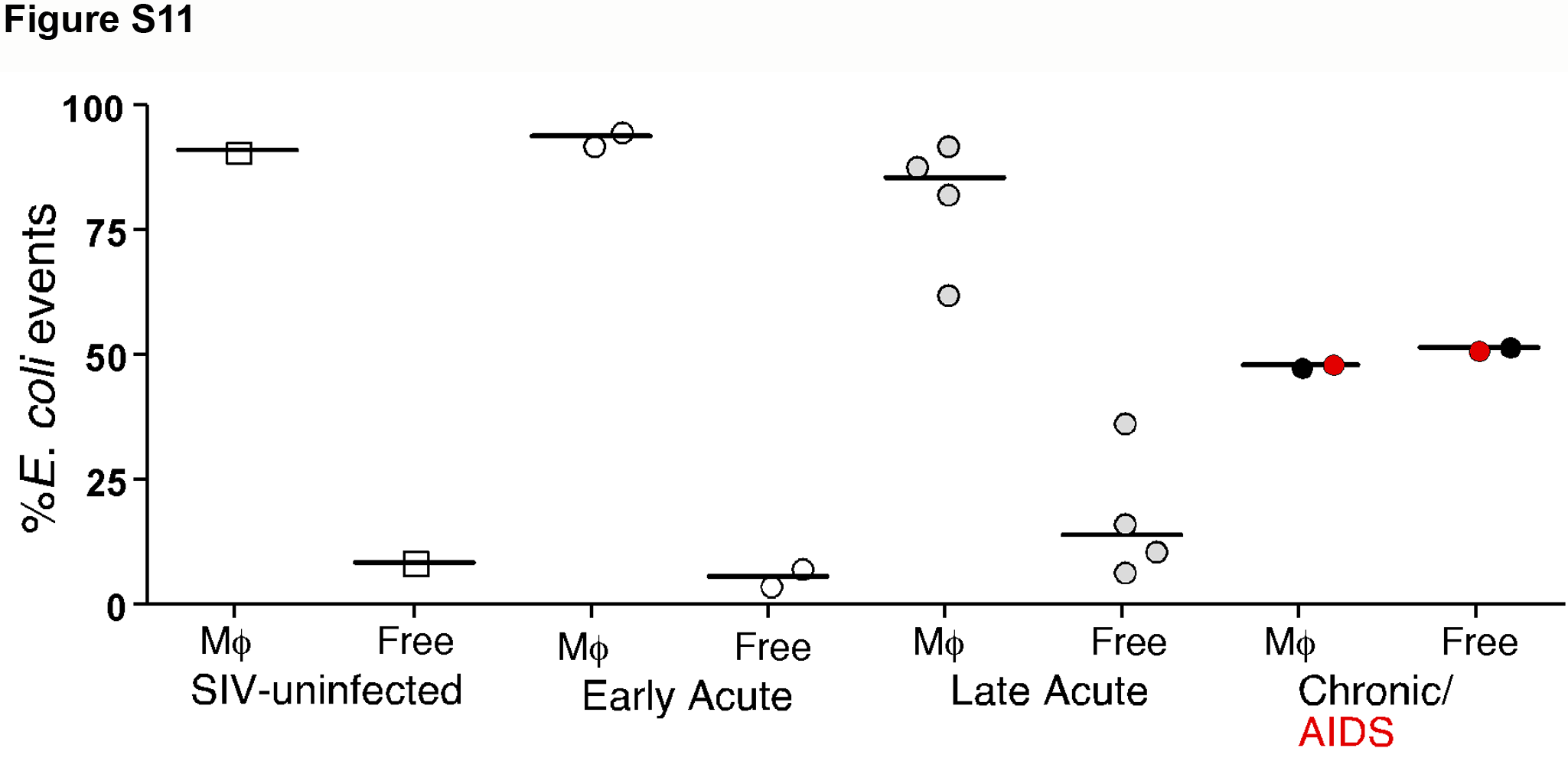

Supplement: Figure S11 — Quantitative image analysis of the frequency of HAM56+ macrophages that are E. coli + compared to the proportion of HAM56− E.coli + events in SIV-uninfected and acutely and chronically SIV-infected RMs. Note that throughout the acute phase of infection (1–21 dpi), most microbial products are within or associated with macrophages, however, starting at 28 dpi and extending into the chronic stage macrophages, although still abundant in the GI tract, become progressively inefficient/dysfunction in their ability to bind and/or phagocytose microbial products that cross the epithelial barrier. (0.13 MB TIF) [file ppat.1001052.s011.tif]
